# Chert outcrops differentiation by means of low-field NMR relaxometry

**DOI:** 10.1038/s41598-024-75945-6

**Published:** 2024-10-25

**Authors:** Michał Fajt, Weronika Mazur-Rosmus, Anna Stefańska, Alicja Kochman, Artur T. Krzyżak

**Affiliations:** Faculty of Geology, Geophysics and Environmental Protection, AGH University of Krakow, al. Adama Mickiewicza 30, Krakow, 30-059 Poland

**Keywords:** Bedded cherts, Nodular cherts, Flints, Relaxometry, PCA, LF-NMR, Solid Earth sciences, Physics

## Abstract

Siliceous rocks served as raw materials in the production of stone tools from the Middle Paleolithic onwards. Due to migration, the provenance of archaeological artefacts can differ from their natural outcrop location. The aim of this work was the application of 1D and 2D low-field nuclear magnetic resonance (LF-NMR) relaxometry to distinguish cherts by their original source. Herein, bedded cherts and accompanying nodular cherts coming from three different outcrops of Kraków-Częstochowa Upland were investigated. 1D and 2D (*T*_*1*_*-T*_*2*_*)* experiments of water-saturated and dry rock sample states delivered *T*_*1*_, *T*_*2*_ times and *T*_*1*_*/T*_*2*_ ratios of distinct hydrogen populations – parameters sensitive to pore size, surface properties, and hydrogen bonding length. In-depth analysis of NMR data showed substantial differences in the porosity, pore surface and pore structure properties of investigated chert samples tested in the three different saturation levels (100% water-saturated, dried and differential). Finally, principal component analysis (PCA) was performed to reduce the number of correlations obtained and highlight the most important NMR properties specific to the particular outcrop localization."Please check captured corresponding author email if correct.""The email is correct"

**Supplementary Information:**

The online version contains supplementary material available at 10.1038/s41598-024-75945-6.

## Introduction

Accurately determining the source of siliceous artefacts and establishing links between them and archaeological discoveries seems to be crucial in the context of studying the migration of prehistoric cultures^1–6^. Understanding the origin of silica raw materials in prehistoric communities could potentially shed new light on prehistoric trade networks and community interactions. However, there is no standardized, objective methodology that utilizes nondestructive techniques to investigate the petrophysical, mineralogical, and geochemical characteristics of chert samples for their natural outcrop differentianion^2,3^.

The Upper Jurassic sediments of the Kraków-Częstochowa Upland (KCU) have attracted attention from both geologists and archaeologists due to the presence of siliceous rocks, as noted in previous studies^1,2^. However the criteria used in typological classifications of artefacts from archaeological sites in Central-Eastern Europe are subjective, ambiguous, and lack a connection to the geological context^1^. The artefacts are often classified based on macroscopic features that are not precisely defined. Therefore, the artefacts made of siliceous raw material from one outcrop may be classified as belonging to different regions of origin of the raw material, or macroscopically similar artefacts from different regions may be classified as the same variety.

Previous attempts to identify the sources of archaeological artefacts have primarily relied on macroscopic characteristics such as colour diversity, shape variations, cortex features, and width measurements. However, these efforts predominantly focused on stone inventories from archaeological sites, neglecting a comprehensive characterization of siliceous rocks in their natural outcrops^1,2^. Furthermore, it is worth highlighting that many petrophysical research methods, like Nuclear Magnetic Resonance (NMR), have been underutilized in archaeological investigations, despite the wealth of information they can provide.

The NMR method has been widely used to study rock cores^1^. H relaxometry in low-field NMR (LF-NMR) has been repeatedly applied to investigate the porosity and permeability of carbonates^7^, shales^8–11^ and recently, even cherts^12^ commonly thought as non-porous. LF-NMR has shown the capability to investigate the pore structure as well as pore surface processes and surface features. Therefore non-invasive LF-NMR method can be used to identify and describe the total porosity, including open as well as closed porosity, in the full-size range from nanoporosity to macroporosity and chemically bound hydrogens (mainly hydroxyl groups). This is possible due to the specificity of the method (sensitivity to all hydrogen species), the experimental setup (dedicated radiofrequency coils enabling very short echo time, *TE*, and hence, the registration of very short *T*_*2*_s, and low magnetic field) and measurements in different water saturation states of a sample. LF-NMR can also deliver more detailed information about the sample, such as free and bound fluid content, pore size distribution, surface processes (adsorption), and chemical features (bond lengths of a bonded fraction) without the need to destroy it^13^.

A previous study has shown^12^, that one- (1D) and two-dimensional (2D) LF-NMR was able to distinguish between bedded cherts and nodular cherts from the Upper Jurassic sediments (of the KCU region) based on the differences in porosity, even though the total porosity did not exceed 2%. This showed that those two types of cherts bear their genetic information enclosed in very specific porosity features that LF-NMR is able to detect. This promising result has prompted a wider application of LF-NMR in more challenging studies of siliceous rocks. In this paper, the potential of LF-NMR to classify bedded and nodular cherts by outcrop based on NMR relaxometry analysis was investigated.

### Geological background

The siliceous rocks present in the Upper Jurassic limestones of the Southern part of the KCU belong to the microbial-sponge megafacies, whose outcrops span from Portugal to the Caucasus region. A distinctive feature of these formations is the significant lithological uniformity observed across extensive areas^2^. KCU is situated in the Silesian-Kraków Homocline, built up of Triassic, Jurassic and Cretaceous sedimentary rocks. The Upper Jurassic sediments comprise massive facies, bedded facies, and submarine gravity flows^14–18^.

**Fig. 1:**
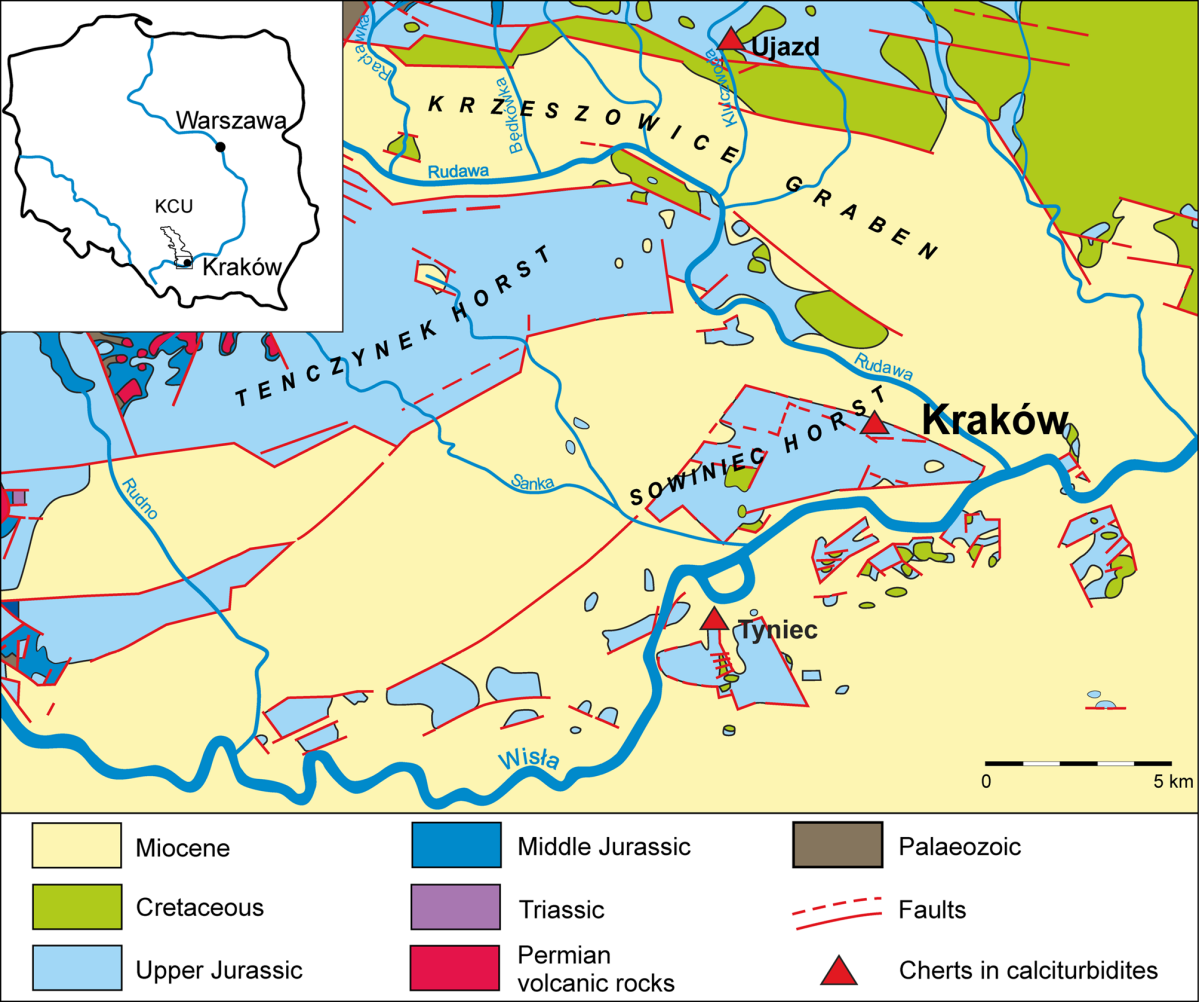
Location of sampling against the bedrock geological map^30^.

Three silicification types and products were distinguished in these facies: nodular cherts, bedded cherts, and epigenetic siliceous rocks which are products of synsedimentary, early-to-late-diagenetic, or epigenetic processes^1,2,14,15,18–28^. Many factors have influenced silicification processes in the KCU region: carbonate host-rock properties, including facies and microfacies, mineralogy, porosity and chemical composition. The genesis of silicification types is not entirely understood and it is the subject of various studies^1,2,18,25–29^. However, only nodular cherts and bedded cherts reveal a clear relationship to the limestone facies in which they occur, in contrast to epigenetic siliceous rocks whose occurrence is not clearly related to any limestones^2^. A characteristic feature of all bedded chert outcrops is their occurrence near the margins of tectonic grabens (Fig. 1) and they have already been described several times in the literature^2,12,20,24,29^.

The bedded cherts occur in an outcrop in Ujazd located near the northern edge of the Krzeszowice Graben, in a disused quarry at Sowiniec Horst and in an exposure in the eastern part of Wielkanoc Quarry on the Tyniec Horst (Fig. 1). The bedded cherts form layers up to half a meter thick and with lengths up to a few meters in calciturbidites which represent a gravity flow deposits from the top of the Upper Jurassic (Oxfordian/Kimmeridgian turn) profile from the southern part of KCU^1,2,18,24^. In the Ujazd outcrop, in addition to bedded cherts, horizons of nodular cherts arranged parallelly to the bedded plane occur. It can be discerned that characteristic concentric growth layers are present in bedded cherts. In contrast to the other siliceous rocks, bedded cherts are characterized by normal fractional grading, the absence of relics of non-silicified limestones, and silicified macrofauna^1^.

## Materials and methods

### Rock core samples

**Fig. 2:**
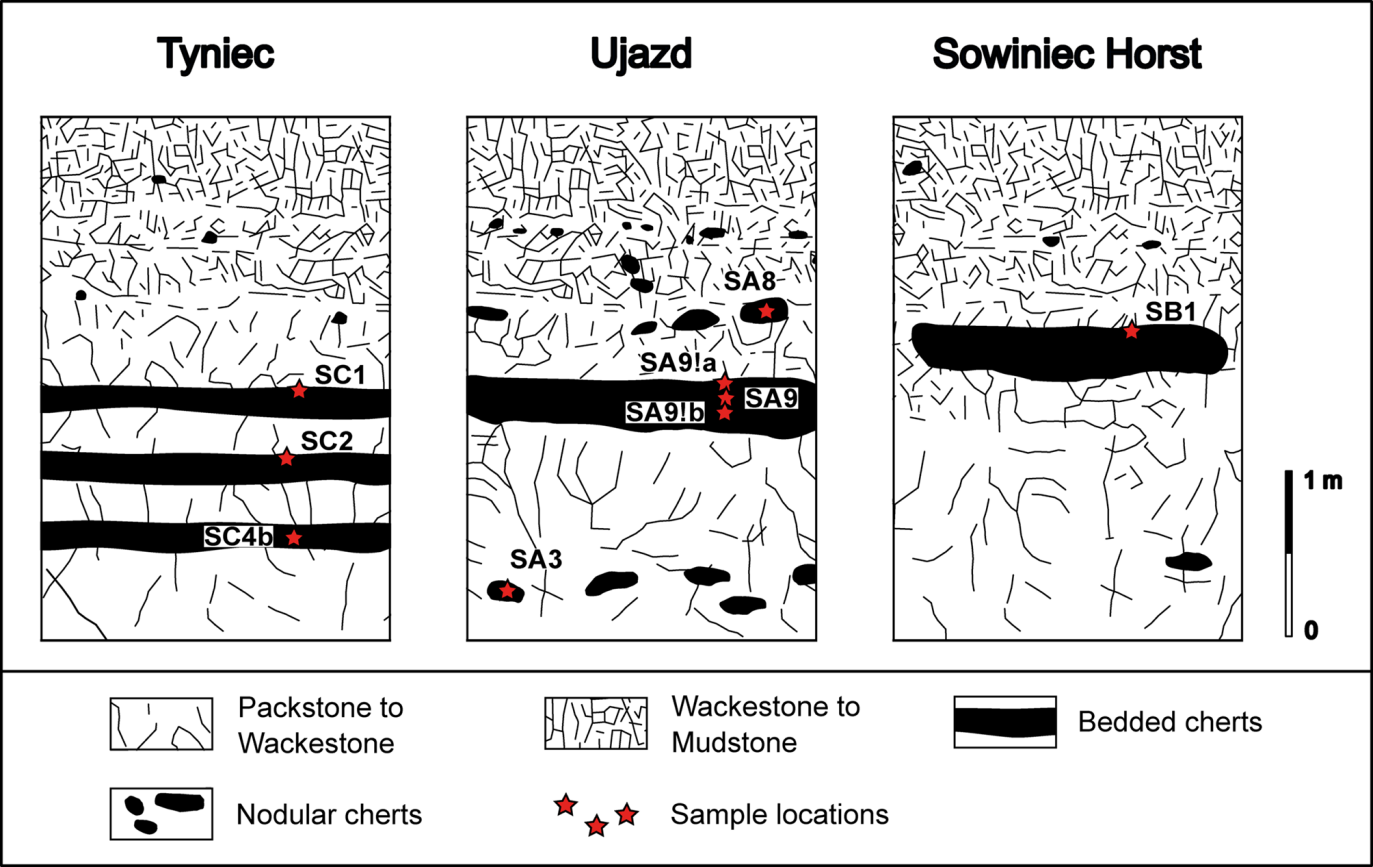
Sketches showing sampling locations in bedded and nodular cherts horizons of analyzed calciturbidite outcrops at Tyniec, Ujazd and Sowiniec Horst.

In total, 9 rock cores were cut from the samples collected from 3 outcrops located in the southern KCU. SB1 was a core of bedded chert from Sowiniec Horst; SA9, SA9!a, SA9!b were cores of bedded cherts and SA3, SA8 were cores of nodular cherts from Ujazd, samples SC1, SC2, and SC4b were bedded cherts cores from the Wielkanoc Quarry at Tyniec (Fig. 1). Sample SB1 was collected from the outer part of the bedded cherts layer from the outcrop of the Sowiniec Horst. Samples SA9 and SA9!a were sampled from the outer, while SA9!b was from the inner part of the bedded cherts layer from the Ujazd outcrop. In Tyniec 3 layers of bedded cherts occur. A sample was taken from each layer. Sample SC4b was from the inner part of the lowest bedded cherts layer, SC2 from the outer part of the middle layer, and SC1 from the outer part of the top layer (Fig. 2). The colours of the bedded chert layers vary from creamy, grey or brownish to dark-creamy, while at the same time, the colours of the inner parts of the chert beds are generally darker and grade outside to brighter shades.

As for nodular cherts: sample SA3 was taken from beneath a layer of bedded cherts and SA8 from above a layer of bedded cherts in the Ujazd outcrop (Fig. 2). The colours of nodular cherts vary from grey to dark grey and black. A more detailed analysis of the macro- and microscopic features of cherts from the studied outcrops of the KCU region is described in previous studies^1,2^.

### Chemical analyses

Major chemical components were determined using lithium metaborate/tetraborate fusion- ICP (Suplementary Table S1 and Table 2 containing components relevant in this study). Samples were prepared and analyzed in a batch system. Each batch contains a method reagent blank, certified reference material and 6% replicates. Samples were mixed with a flux of lithium metaborate and lithium tetraborate and fused in an induction furnace. The molten melt was immediately poured into a solution of 5% nitric acid containing an internal standard and mixed continuously until completely dissolved oxides and selected trace elements on an ICP. The geochemical analyses were carried out at Activation Laboratories Ltd. in Ancaster, Canada.

### Mercury injection capillary pressure (MICP)

MICP measurements were conducted using the Micromeritics AutoPore IV 9520 mercury porosimeter. Rock core samples were crushed and dried at 105 °C for 24 h dried to remove moisture from the pore spaces and then cooled to room temperature (API-RP 40/98, ASTM-D4404-10, 2010). The analysis was carried out assuming the sample evacuation to 50 µmHg (6.67 Pa) for 2 min and the equilibrium time of 10 s. Pressure was measured at 82 points in the range of 2–60 000 psi (0.01-413.69 MPa). MICP delivered surface-area-to-volume ratios (S/V) of a probed pore space (Table 2).

### Magnetic susceptibility measurements

Magnetic susceptibility measurements were performed using a Bartington MS2 meter (Bartington Instruments Ltd.) in combination with a sensor for laboratory magnetic susceptibility measurements. The role of the sensor is played here by a thermally stable oscillator and an induction coil, which affects the oscillator frequency. In a situation where there are no bodies with magnetic properties near the coil, the oscillator frequency depends only on the magnetic permeability of the air. If a sample with magnetic properties is near the coil, the magnetic permeability of this sample modulates the oscillation frequency. The device performs its own calibration of the oscillation frequency, which is converted to magnetic susceptibility. Measured mass (χ_m_) and volume (*χ*_*sample*_) magnetic susceptibilities are shown in Table 2.

###  Nuclear magnetic resonance

#### Theory

It has been shown that homonuclear dipolar couplings and scalar effects are responsible for most of the NMR relaxation mechanisms of fluids in rock^31–33^. The former deals with the interaction of proton spins in a liquid or bound on a rock surface. For the latter, the relaxation time is then determined by the interaction of water protons and the magnetic moment of ions having unpaired electrons^32,33^.

The strength of homonuclear dipolar couplings can be assessed by measuring longitudinal (*T*_*1*_) and transverse (*T*_*2*_) relaxation times, which are described by the following formulas:^32^

1 $$\:\frac{1}{{T}_{1}}=2 C\left[\frac{2\tau\:}{1+{\omega\:}^{2}{\tau\:}^{2}}+\frac{8\tau\:}{1+4{\omega\:}^{2}{\tau\:}^{2}}\right],$$

2 $$\:\frac{1}{{T}_{2}}=C\left[6\tau\:+\frac{10\tau\:}{1+{\omega\:}^{2}{\tau\:}^{2}}+\frac{4\tau\:}{1+4{\omega\:}^{2}{\tau\:}^{2}}\right],$$

where $\:\tau\:$ is the correlation time of dipolar interactions, $\:\omega\:$ is the Larmor frequency and $\:C$ is a constant. The above formulas show the dependency of relaxation times on the single correlation time for simplicity (in reality, we deal with two correlation times of rotational and tumbling motions of a molecule). They can help to analyze the values of experimental relaxation times on a molecular level. For example, the correlation time increases with the immobilization, and for nonmobile species one gets $\:\omega\:\tau\:\gg\:1$ and consequently $\:\frac{{T}_{1}}{{T}_{2}}\sim\:{\omega\:}^{2}{\tau\:}^{2}\gg\:1$. Therefore, based on the ratio it is possible to assess the mobility of a given hydrogen population.

Moreover^1^, ^1^H standard Larmor frequency changes for complex chemical compounds containing hydrogen due to the changing chemical environment. The change of $\:\omega\:$ for those hydrogens in chemical structures is called a chemical shift ($\:{\delta\:}_{H}$). For example, for hydroxyl groups (OH) relevant in this study, it is linearly dependent on the infrared vibrational frequency ($\:{\nu\:}_{OH}$) of the species in the given $\:{\nu\:}_{OH}$ intervals (the dependence is non-linear in the whole range of stretching frequencies). The example of empirical formulas connecting $\:{\delta\:}_{H}$ with $\:{\nu\:}_{OH}$ are:^34^

3 $$\:{\delta\:}_{H}=57.1-0.0147{\nu\:}_{OH},$$

for surface hydroxyls and

4 $$\:{\delta\:}_{H}=37.7-0.0092{\nu\:}_{OH},$$

for hydrogen-bonded protons (for example of the hydrate water in solids). For bonded protons, the empirical relation for $\:{\delta\:}_{H}$ in connection to the bond length was also found:

5 $$\:{\delta\:}_{H}=4.65\cdot\:{r}_{OH}^{-1}-17.4,$$

Therefore, the change in relaxation times is also dependent on the chemical shift (local molecular environment of^1^ ^1^H) and the bond length as shown in the case of hydroxyls.

Macroscopically, the observed (experimental) $\:{T}_{1}$ and $\:{T}_{2}$ relaxation times are the effective values depending on the dephasing due to bulk liquid interactions ($\:{T}_{1/2,\:bulk}$), surface interactions ($\:{T}_{1/2,\:surface}$) and diffusion due to gradients induced by the differences in magnetic susceptibilities ($\:{T}_{2,\:diffusion}$):^35^

6 $$\:\frac{1}{{T}_{1}}=\frac{1}{{T}_{1,\:bulk}}+\frac{1}{{T}_{1,\:surface}},$$

7 $$\:\frac{1}{{T}_{2}}=\frac{1}{{T}_{2,\:bulk}}+\frac{1}{{T}_{2,\:surface}}+\frac{1}{{T}_{2,\:diffusion}}\approx\:\frac{298\eta\:}{3{T}_{K}}+\frac{{\rho\:}_{2}S}{V}+\frac{{\left(\gamma\:{G}_{ind}TE\right)}^{2}D}{12},$$

where $$\:{T}_{1/2,\:bulk}$$ is relaxation for water unaffected by sink mechanisms, $$\:{T}_{K}$$ (K) is temperature and $$\:\eta\:$$ (Pa∙s) is viscosity, $$\:{\rho\:}_{1/2}$$ (m/s) is surface relaxivity (the strength of the surface to cause additional spins dephasing and echo amplitude decrease) and $$\:\frac{S}{V}$$ (1/m) is surface-area to volume ratio of a confining geometry, $$\:D$$ (m^2^/s) is diffusion coefficient, $$\:\gamma\:$$ (1/T∙s) is the gyromagnetic ratio, $$\:TE$$ (s) is echo time and $$\:{G}_{ind}$$ (T/m) is the internal constant gradient equal to $$\:\frac{{\Delta\:}\chi\:{B}_{0}}{d}$$, where $$\:{B}_{0}$$ (T) is the external magnetic field induction, $$\:d$$ (m) is pore diameter and $$\:{\Delta\:}\chi\:$$ the difference between volume magnetic susceptibilities of saturating fluid ($$\:\chi\:$$$$\:{}_{fluid}$$) and a sample’s matrix ($$\:{\chi\:}_{sample}$$). Based on those formulas, pore sizes and surface properties can be assessed. For example, in a system, where surface relaxation dominates the ratio of $$\:\frac{{T}_{1}}{{T}_{2}}\approx\:\frac{{T}_{1,\:surface}}{{T}_{2,\:surface}}$$ reflects the adsorption energy^36^.

#### LF-NMR experiments

Each of the samples was measured in the three water saturation states in the following order: (1) dried (for 12 h at 200^o^C); (2) saturated (for 24 h in distilled water under vacuum and room temperature conditions). Saturated samples were protected with a residual amount of plastic foil (0,06 g) to avoid evaporation of the absorbed water. In addition, samples were examined in a (3) differential state, which is an artificial saturation state obtained via subtracting dry sample raw data from the saturated sample raw data. In this approach, only movable water was characterized, which informed us about open porosity.

1D-*T*_*1*_, 1D-*T*_*2*_ and *T*_*1*_-*T*_*2*_ NMR experiments were carried out for samples in dry (D), saturated (S) and differential (SD) saturation states on a 2 MHz Magritek Rock Core Analyzer (Aachen, Germany) applying Inversion Recovery (IR), Carr − Purcell − Meiboom − Gill (CPMG) and combined IR-CPMG sequences, respectively. Key parameters are shown in Table 1. 1D distributions were calculated in Prospa software (Magritek, Aachen, Germany) using Inverse Laplace Transform (ILT) applying the Lawson and Hanson method and *T*_*1*_ − *T*_*2*_ correlation maps applying the FISTA algorithm^37^. From all 1D *T*_*2*_ distributions cumulative porosities (*φ*_*i*_) were calculated (Supplementary Table S4), where *i* indicated a saturation state. By using standard LF-NMR protocol^9^ on saturated and dry samples data *T*_*2cutoff*_ values (*T*_*2*_ of the boundary between irreducible and movable water) were obtained, thus main porosity parameters: bulk-volume irreducible (*BVI*), free-fluid index (*FFI*), saturated water irreducible (*SWI*) and Total Porosity could be estimated (Supplementary Table S3). 1D-*T*_*2*_ (*T*_*2lm*_) and 1D-*T*_*1*_ (*T*_*1lm*_) logarithmic means of distributions were also determined. Peaks visible on 1D distributions were numbered X_i_ starting from the lowest relaxation time, where X indicated a saturation state (d, s, sd for 1D-*T*_*1*_ and D, S, SD for 1D-*T*_*2*_) for which a distribution was obtained, and i = 1, 2, …, *n*, where *n* is a total number of peaks (Supplementary Tables S2 and S4, respectively). Peaks visible on 2D distributions were numbered numbered according to the following classification: (1) *T*_*2*_ ~ 0,05 ms, *T*_*1*_ ~ 20 ms; (2) *T*_*2*_ ~ 0,1 ms, *T*_*1*_ ~ 100 ms; (3) *T*_*2*_ ~ 0,1 ms, *T*_*1*_ ~ 10 ms; (4) *T*_*2*_ ~ 1 ms, *T*_*1*_ ~ 1 ms; (5) *T*_*2*_ ~ 1 ms, *T*_*1*_ ~ 10 ms; (6) *T*_*2*_ ~ 1 ms, *T*_*1*_ ~ 100 ms; (7) *T*_*2*_ ~ 20 ms, *T*_*1*_ ~ 200 ms; (8) *T*_*2*_ ~ 20 ms, *T*_*1*_ ~ 300–1600 ms; (9) *T*_*2*_ ~ 130–200 ms, *T*_*1*_ ~ 400–2000 ms (Supplementary Table S5).

**Table 1:** Protocol parameters used in LF-NMR experiments. RT is the inter-experiment time (time between subsequent π-pulses), TE is the echo time, NoE is the number of echoes in the echo train, Min-Max delay is the range of the separation times between π and π/2-pulses in IR sequence from minimum to maximum, *τ*_*min*_-*τ*_*max*_ is the range of separation times between π/2 and π-pulses from minimum to maximum in CPMG sequence, NoS is number of scans, steps is a number of time steps (delay and τ for IR and CPMG, respectively) and α is ILT smoothing factor.

Experiment	TR (ms)	TE (µs)	NoE	Min-Max delay (ms)	τ_min_-τ_max_ (ms)	NoS	Steps	α
1D-*T*_*1*_	5000	-	-	0,05-5000	-	24	30	1
1D-*T*_*2*_	1500	60	10 000	-	-	512	-	0,6
*T*_*1*_-*T*_*2*_	1500	60	10 000	0,05-5000	0,1-5000	64	30	1

#### Pore size distribution (PSD)

PSDs were estimated for differential state distributions, since only them represent the open (effective) pore space and therefore correspond to reference MICP measurements in which only the penetrable pore throat system is observed.

Firstly, surface relaxivity, $\:{\rho\:}_{2}$, was estimated (Supplementary Table S3) using the commonly used approximation $\:\frac{1}{{T}_{2}}\approx\:\frac{1}{{T}_{2,\:surface}}$. This is usually valid for rocks with none or weak paramagnetic doping, for which diffusional component can be minimized by the short echo time, *TE* (Table 1) and the application of the small magnetic field $\:{B}_{0}$ of 0.05 T. Since the bulk relaxation, $\:{T}_{2,\:bulk}$ is much larger than registered relaxation times *T*_*2*_, Eq. (7) can be reduced to surface component only yielding approximate surface relaxation:^38–40^

8 $$\:{\rho\:}_{2}={\left({T}_{2,\:surface}\cdot\:\frac{S}{V}\right)}^{-1},$$

where $\:{T}_{2,\:surface}={T}_{2}$, and in application logarithmic mean of *T*_*2*_ distribution in differential state was used to obtain the effective value of surface relaxivity for the whole PSD^41^.

Secondly, we accounted for the fact that diffusion relaxation occurs, and water molecules are less mobile in nanopores, which causes the decrease of diffusivity. This was made by substituting constant diffusion coefficient in Eq. (7) with function *D*(*d*), where *D* is diffusion coefficient and *d* is pore diameter. To find *D*(*d*) function regression analysis was performed and based on literature values of diffusion coefficient^42–47^. logistic function was fitted (Supplementary Fig. 1). It is worth noting that the choice of *D*(*d*) function did not influence the resulting PSD if it fulfilled the following requirements: (1) *D*(*d* < 1 nm) = 0.045∙10^−9^ m^2^/s; (2) maximal *D*(*d*) = *D*_*bulk*_, where *D*_*bulk*_ is a diffusion coefficient of bulk water.

Finally, PSD was calculated in MATLAB (The MathWorks, Inc., Natick, USA) using the following formula obtained on the basis of Eq. (7):^12^

9 $$\:C\cdot{d}^{2}-4\cdot\:{\rho\:}_{2}\cdot\:d-D\left(d\right)\cdot\:F=0,$$

where $\:C=\frac{1}{{T}_{2}}-\frac{1}{{T}_{2,\:bulk}}$, $\:F=\frac{1}{12}{\left(\gamma\:\varDelta\:\chi\:{B}_{0}TE\right)}^{2}$, $\:{T}_{2,\:bulk}=2.2\:s$ and $\:{T}_{2}$ is transverse relaxation time obtained in the experiment.

For comparison, PSDs were calculated using a common approach in which diffusional component was omitted (Supplementary Fig. 4), assuming cylindrical pore shapes, as only valid for obtained surface relaxivity values, using:^12^

10 $$\:{d}_{approx}={4{\rho\:}_{2}\cdot\:T}_{2},$$

The difference between distributions then reflects the influence of diffusion.

#### Application

We wanted to diversify the collected samples as much as possible so that the results of their differentiation would not be biased. For example, the research group included samples of bedded cherts that were taken not only from different outcrops but also from different parts of the bedded chert layers (inner and outer). Thanks to this, it was possible to assess which NMR parameter distinguishes samples based on a given criterion and to find a set of parameters that will divide the entire group of samples depending on the outcrop. Moreover, our goal was to find the reasons for the differences in the obtained relaxation times and other derived NMR parameters for the samples. Because relaxation time in rocks reflects many properties at the same time, the problem is quite complex and requires extensive analysis, which was carried out in the following steps.

**Fig. 3:**
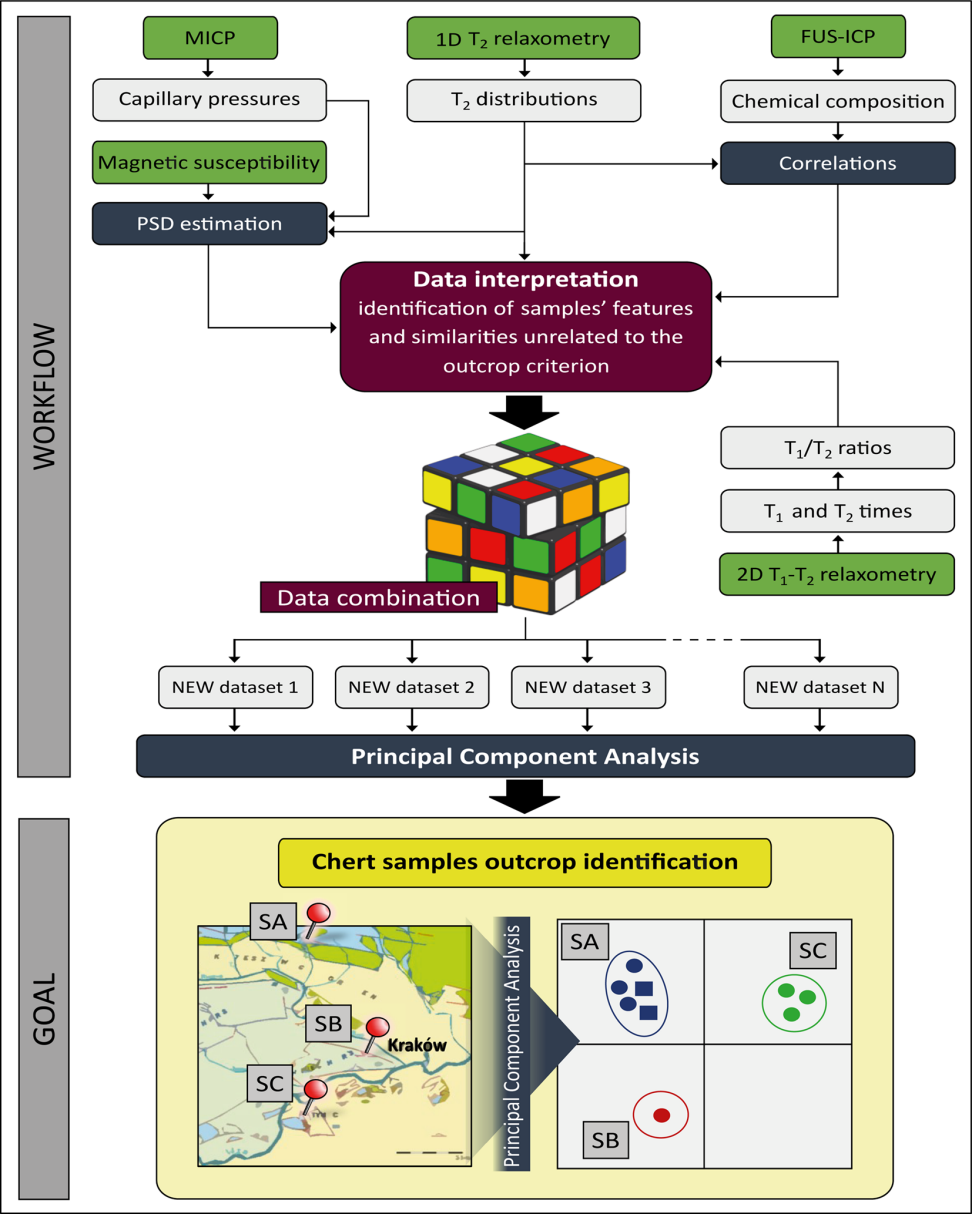
Workflow of LF-NMR methodology and chemical component analysis used to associate studied nodular and bedded chert samples with their sampling outcrop in the KCU area.

First, longitudinal and transverse relaxation experiments were performed, and correlation maps of these processes were measured for dry and saturated samples. Later, it was checked whether there are relationships between geochemical and NMR parameters, which allowed for insight into the extent to which the chemical composition determines the value of a given NMR parameter (for example, how the relaxation time changes depending on the content of ferromagnetic iron in siliceous rock matrix). Then, the relaxometric results were characterized in such a way as to identify the source of a given peak in the 1D and 2D distributions of relaxation times and their differences depending on the sample silicification type (bedded/nodular), outcrop localization and position in chert layer (for bedded cherts). The next step was to determine correlations between relaxometric parameters and the chemical composition of investigated samples, as well as to estimate the pore size distribution (PSD) and examine whether there were sufficient differences to distinguish outcrops. Finally, principal component analysis (PCA) was performed on the various sets of parameters that differed among the outcrops in the above steps to check whether those differences were significant enough for the samples clustering according to their place of origin. PCA is the culmination of analyzing the differences between outcrops in a statistical manner. It highlighted key NMR parameters that determined the grouping of samples from different outcrops, which could speed up the discrimination process in the future by reducing the number of necessary experiments and analysis steps. The described workflow is presented schematically in Fig. 3.

### Porosity data validation

As the reference for NMR porosity determination methods, results of mass-volume measurements obtained during the samples preparation process for LF-NMR experiments were used. Mass-volume porosity (φ_mass−vol_) was calculated by comparing the saturated density of a sample (mass of 100% water-saturated sample per unit volume) to its bulk density (the mass of a dry sample per unit volume), with respect to saturating fluid (water) density, according to Eq. (11):

11 $$\:{\varPhi\:}_{mass-vol}=\frac{{m}_{sat}-{m}_{dry}}{{\rho\:}_{w}{V}_{sample}}\cdot\:100,$$

where m_sat_ and m_dry_ are the mass of saturated and dried samples respectively (g); ρ_w_ is the density of water (g/cm^3^); and V_sample_ is the bulk volume of the sample in dry state (cm^3^).

The results of mass-volume porosity measurements are shown in Supplementary Table S3. Results of cross-validation between mass-volume porosity and open porosity derived from differential distributions are shown in Supplementary Figure S6.

### Principal component analysis (PCA)

The PCA was performed using the PQStat software (Poznań, Poland). It consists of transforming the set of primary variables into a new set of variables (principal components) based on statistical information (the correlation between primary variables). This analysis is used to reduce the number of variables and simplify the process of finding samples with similar characteristics. A more detailed description of the calculation and interpretation methods can be found in^12^. PCA was carried out for three data sets: (1) *T*_*2*_ times of all peaks visible in the 1D-*T*_*2*_ distributions of the dry and saturated samples numbered depending on the region of occurrence, porosity for a given saturation state and *T*_*2cutoff*_ ; (2) *T*_*2*_ times of all hydrogen populations visible in the 2D *T*_*1*_-*T*_*2*_ correlation maps obtained from saturated samples numbered depending on the region of occurrence (see Sect. 2.3); (3) *T*_*2*_ times of all hydrogen populations visible in the maps obtained from saturated samples and associated *T*_*1*_*/T*_*2*_ ratios, and contents of SiO_2_ and CaO as dominant compounds. Additionally, three supplementary PCA were performed (Supplementary Figure S5) for the following datasets: (S1) chemical composition; (S2) chemical composition and parameters from a standard NMR porosity analysis protocol *T*_*2cutoff*,_*BVI* and *FFI*; (S3) *T*_*2*_ times from maps of saturated samples, and corresponding *T*_*1*_/*T*_*2*_ ratios, similarly to (3) but with standard chemical parameters used for chert samples differentiation: *Fe*_*2*_*O*_*3*_*/TiO*_*2*_ and *Al*_*2*_*O*_*3*_*/(Al*_*2*_*O*_*3*_ *+ Fe*_*2*_*O*_*3*_*)*.^48^

## Results and discussion

The bedded cherts under analysis were composed mainly of SiO_2_, CaO and Fe_2_O_3_ (significant components from the point of view of NMR relaxometry; Table 2). The nodular cherts (SA3 and SA8) had a higher content of SiO_2_ and Fe_2_O_3_ on average than bedded cherts. All samples, besides SA9!a appear to exhibit chemical characteristics similar to the Ocean Ridge environment of deposition (Supplementary Figure S3b) with a strong influence of hydrothermal activity in the silicification process (Supplementary Figure S3d). Sample SA9!a undergone a lower degree of silicification, as evidenced by the lowest SiO_2_ and highest CaO content (Table 2). What is noteworthy is that the other commonly used charts of chemical element ratios^48^ have proved ineffective in attempting any differentiation of the studied samples (Supplementary Figure S3a, c).

**Table 2:** Major element contents (%) in the bedded and nodular cherts with MICP delivered surface-area-to-volume ratios (S/V) and measured mass (χm) and volume (χsample) magnetic susceptibilities.

Sample	Outcrop	Host rock	SiO_2_	Fe_2_O_3_	CaO		S/V (µm^−1^)	χ_m_ (m^3^/kg)	χ_sample_
SB1	Sowiniec Horst	calciturbidites	92,76	0,61	3,58		500	-4.57E-09	-1.17E-05
SA3	Ujazd	beneath a layer of bedded chert in calciturbidites	99,02	1,03	0,10		639	-2.29E-09	-5.88E-06
SA8		above a layer of bedded chert in calciturbidites	97,94	0,96	0,05		659	-1.43E-09	-3.69E-06
SA9		calciturbidites	69,65	0,21	16,94		460	-2.5E-09	-6.24E-06
SA9!a		calciturbidites	58,74	0,14	23,05		589	-1.91E-09	-4.72E-06
SA9!b		calciturbidites	91,17	0,74	4,05		366	-4.42E-09	-1.12E-05
SC1	Tyniec	calciturbidites	87,11	0,52	7,08		500	-4.08E-09	-1.05E-05
SC2		calciturbidites	83,47	0,48	9,32		375	-2.54E-09	-6.44E-06
SC4b		calciturbidites	86,44	0,63	7,14		729	-2.66E-09	-6.83E-06

As described above, discriminant diagrams of the major chemical components as well as geochemical results have proven ineffective at distinguishing between the different chert outcrop locations. However, these findings further confirm that hydrothermal activity related to extensional tectonics in the Late Jurassic had a great influence on silicification processes in the calciturbidite sediments of the KCU area^2,26,28,49^.

### Outcrops differentiation based on one-dimensional techniques

#### Correlation between chemical composition and NMR parameters

**Fig. 4:**
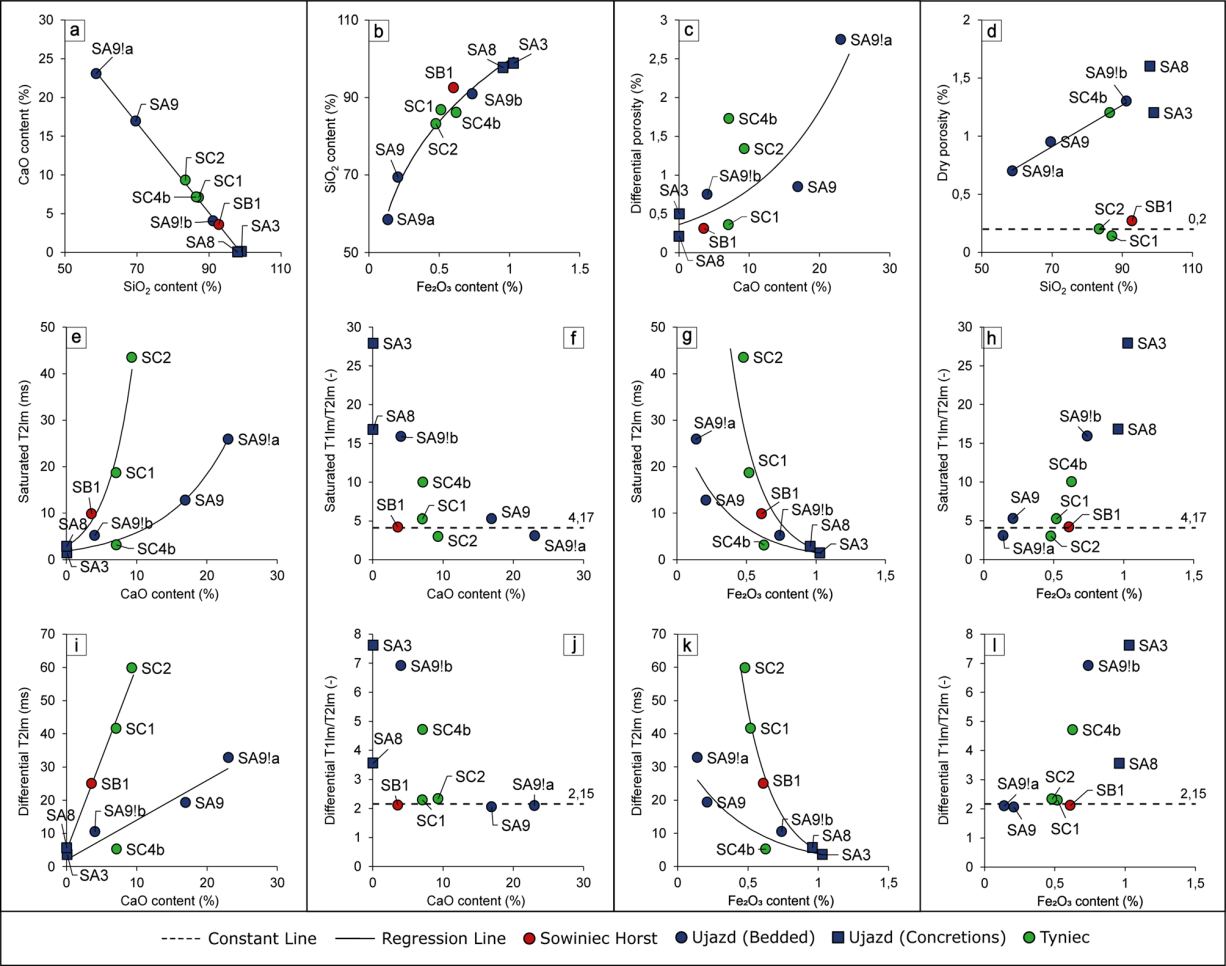
Correlation between chemical compounds content (a, b) and the dependency of NMR parameters on chemical compounds content (c-l).

**Fig. 5:**
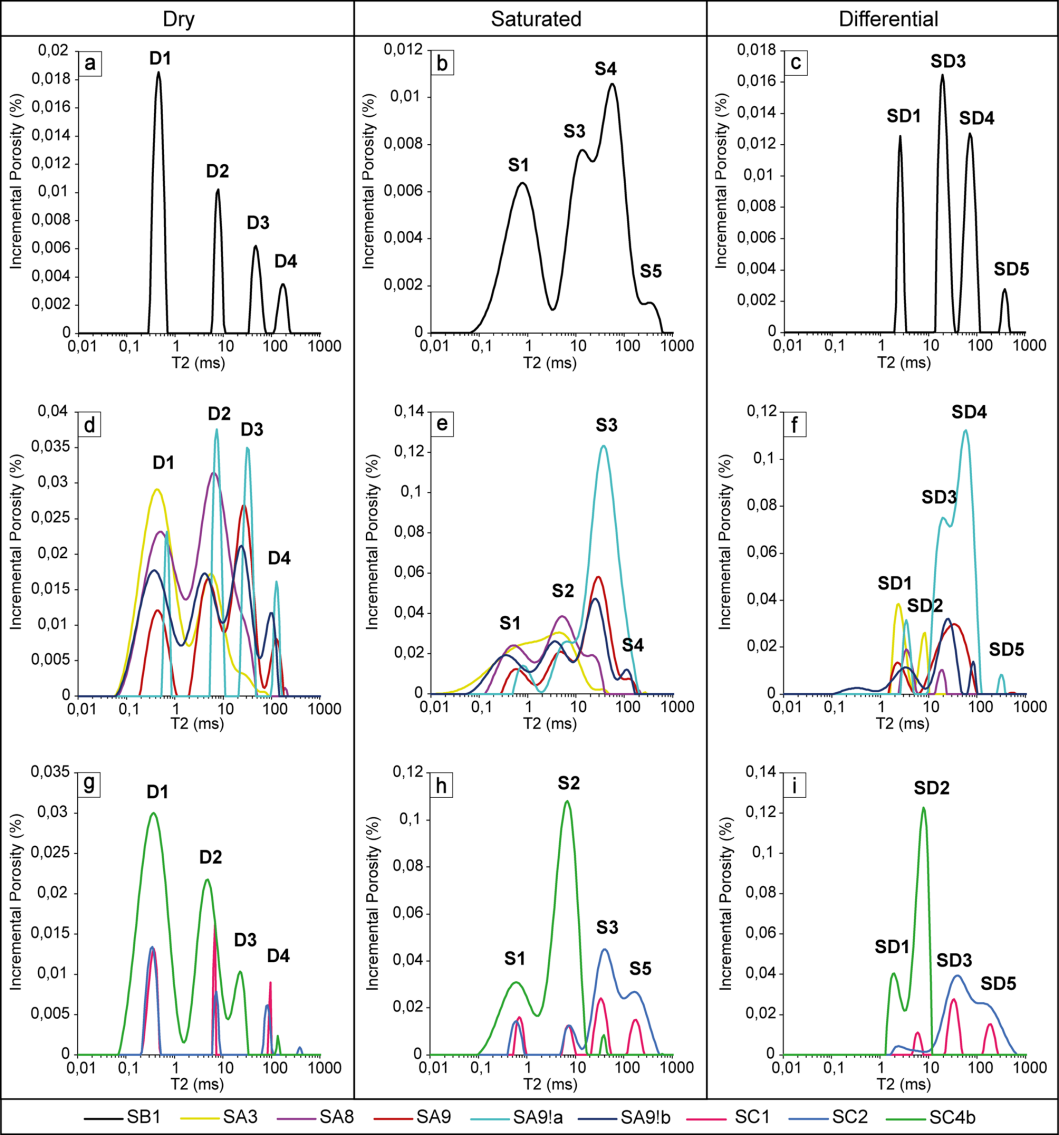
T_2_ distributions of dry (first column), saturated (second column) and differential (third column) data of investigated chert samples with the division to three outcrops: Sowiniec Horst (sample SB1, panels a-c), Ujazd (samples SA3, SA8, SA9, SA9!a, SA9!b, panels d-f) and Tyniec (samples SC1, SC2, SC4b, panels g-i).

1D NMR relaxometry of the samples delivered *T*_*1*_ (Supplementary Figure S2) and *T*_*2*_ times distributions (Fig. 4), from which besides the *T*_*1*_s and *T*_*2*_s of the observed peaks, logarithmic means, porosities (from 1D-*T*_*2*_ experiments) and the ratio of *T*_*1lm*_/*T*_*2lm*_ were calculated (Supplementary Table S4). Distributions for the rocks in the saturated state correspond to the total porosity, in the differential state to the open porosity, and in the dry state to the closed porosity. Porosity represented by saturated. and to a greater extent, dry states is influenced by signals from the chemically bound hydrogen and adsorbed water signals. The ratio of *T*_*1lm*_/*T*_*2lm*_ is a parameter which can inform us about the effective adsorption strength in the sample^36^. According to Fleury and Romero-Sarmiento^50^, water in pores has *T*_*1*_/*T*_*2*_ ~ 2 and the stronger bounding of hydrogen (smaller pores, viscous fluid, adsorbed water, chemically bound hydrogen), the higher the ratio is observed. Porosities, *T*_*2lm*_s and *T*_*1lm*_/*T*_*2lm*_ were checked on the correlation with chemical compound content and the following observations were made:

- dependency between **SiO**_**2**_**and CaO content** (Fig. 5a) **was linear**, while, interestingly, **exponential between SiO**_**2**_**and Fe**_**2**_**O**_**3**_ (Fig. 5b) illustrating lower reduction of the calciturbidite host-rock matrix due to silicification in samples from outer parts of bedded cherts bed and the increasing influence of iron content and silica abundance in all measured cherts samples;
- **differential porosity increased exponentially with CaO content** (Fig. 5c), which indicates that the main part of the open porosity of the studied cherts is related to the residual carbonate content;
- **dry porosity was constant (0.2%) for samples from the outer parts of bedded cherts layers in Tyniec and Sowiniec Horst outcrops**,** while increased linearly with SiO**_**2**_**content for all bedded chert samples from Ujazd and SC4b sample from the inner part of bedded cherts layer in Tyniec outcrop** (Fig. 5d). In low CaO content samples, high dry porosity could be connected to the significant number of inclusions (closed pores and structural hydroxyls). For high CaO content (observed for samples from outer parts of chert beds) it occurs due to irreducible capillary-bound water, which is commonly observed for carbonates^7^. Only samples from the outer parts of bedded cherts layer in Ujazd exhibit an increased number of closed pores in their SiO_2_ matrix showing the unique NMR characteristic of all bedded chert samples from this outcrop.
- ***T***_***2lm***_**from the saturated state increased exponentially with CaO content** (Fig. 5e), while **linearly for the differential state** (as expected for pore bulk water) showing the constant increase in signal from open porosity in bigger pore spaces with increased carbonate content as well as disruption effect of this relationship by the influence of chemically bound hydrogen and adsorbed water information in the signal of a 100% water-saturated sample. (Fig. 5i). At the same time, a lesser effect of the carbonate content on the increase in T_2lm_ in the Ujazd samples is evident, as is a similarly weaker effect of iron content on its decrease;
- both saturated and differential ***T***_***2lm***_**decreased exponentially with Fe**_**2**_**O**_**3**_ (Fig. 5g, k), which can be connected to the influence of silicification over pore size reduction. Also in this case we observe weaker emanation of this phenomenon in all Ujazd samples;
- ***T***_***1lm***_**/*T***_***2lm***_**of saturated and differential states was independent of CaO content** (mean equal to around 4.17 and 2.15, respectively; the lower mean was observed in the differential state, because it reflects movable water in pores, while the saturated state combines also chemically bound hydrogen and adsorbed water signal having higher *T*_*1*_/*T*_*2*_) **except for four samples: nodular cherts and bedded cherts: SA9!b and SC4b** (Fig. 5f, j) (**these samples were cut from the inner parts of the bedded chert layers and had the highest silica content** and their *T*_*1lm*_/*T*_*2lm*_ = 10-27.9 and 3.55–7.61 in the saturated and differential state, respectively). This suggests that high values observed for the samples from the inner part of the bedded chert layer and nodular cherts correspond to tighter voids (based on differential state), strongly adsorbing surface of the crystal lattice and possibly structural hydroxyls (based on saturated state);

It can also be seen that the content of just 3% CaO (excluding outliers) dominates the effective *T*_*1*_/*T*_*2*_ for the sample, probably due to the significantly lower content of porosity in the SiO_2_ matrix. The influence of Fe_2_O_3_ content on *T*_*1lm*_/*T*_*2lm*_ exhibits similar but reversed characteristics as of CaO (Fig. 5h, l). *T*_*1lm*_/*T*_*2lm*_ was constant for the bedded chert samples from the outer parts of bedded chert layers, with Fe_2_O_3_ content up to 0.61%. However, for higher Fe_2_O_3_ content the increase in the *T*_*1lm*_/*T*_*2lm*_ ratio was observed.

#### *T*_*2*_ distributions

On 1D-*T*_*2*_ distributions five different hydrogen populations (peaks) could be distinguished (Fig. 4). They were numbered from 1 to 5 for the following *T*_*2*_ regions: 0,1–1 ms, ~ 1–10 ms, ~ 10–50 ms, ~ 50–150 ms, > 150 ms. In the dry state, all samples were similar in terms of peak positions (Fig. 4, first column). Differences can be seen in peak amplitudes and the number of visible peaks. The main observations according to differences among samples are:

- **SA9!a stood out within its group due to very high CaO content**, which suggests the significant occurrence of carbonate host-rock residues in the sample, that are characterized by bigger voids with higher *T*_*2*_s;
- **SC4b showed a higher abundance of short*T***_***2***_**and a lack of high*T***_***2***_**hydrogen populations** in comparison to other samples from the same outcrop and to the SA9!b sample that also comes from the inner part of the bedded chert layer. Only this sample was so strongly saturated in the tighter spaces with *T*_*2*_ = 1–10 ms (the characteristic regions that are predominantly saturated in nodular cherts are *T*_*2*_ ~ 1 ms and *T*_*2*_ ~ 4–40 ms in bedded cherts, which suggests that SC4b has mixed features of open porosity which are characteristic for nodular and bedded cherts, according to^12^);
- **after water saturation porosity increased 1.1–8.7 times**,** while*T***_***2lm***_**1.2–29 times**: the smallest changes were observed for samples SA3 and SA8 (1.4- and 1.1-times higher porosity and 1.4- and 1.2-times higher *T*_*2lm*_, respectively) pointing to the lowest connectivity of nodular cherts pore space and the highest proportion of closed porosity relative to total porosity;
- **outcrops differed in terms of samples’ dynamic changes in porosity (amount and size**, reflected in porosity and *T*_*2lm*_ change, respectively): the average ratio of saturated and dry porosities for samples within the outcrop groups were equal to 2.1, 2.2 and 4.9 (2.1, 2.9 and 4.9 excluding nodular cherts), while 3.5, 1.6 and 16.2 in case of *T*_*2lm*_ (3.5, 1.7 and 16.2 excluding nodular cherts) for the Sowiniec Horst, Ujazd, and Tyniec, respectively.

#### Pore size distributions

**Fig. 6:**
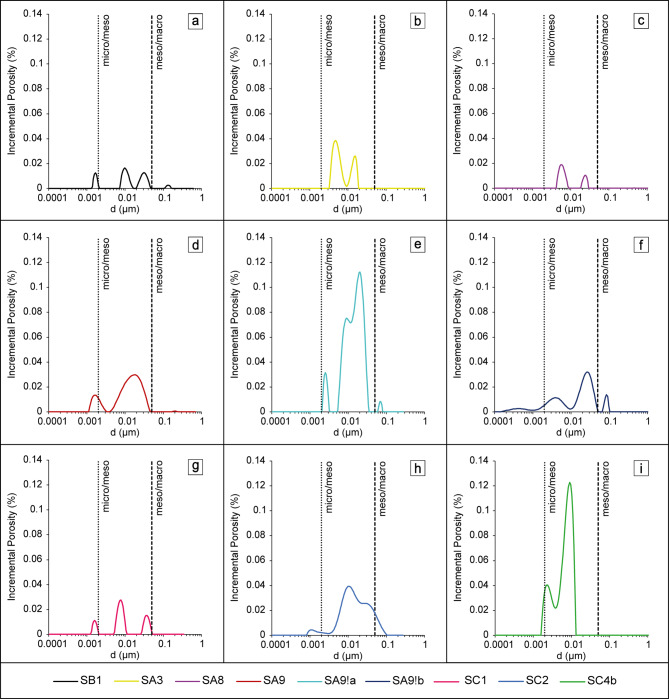
PSD distributions of investigated chert samples.

PSDs were calculated from Eq. (10) using 1D-*T*_*2*_ data from obtained differential distributions and shown in Fig. 6. Next, PSDs were divided into meso- and macroporosity according to IUPAC classification^51^ and geometric averages of pore sizes were calculated (Supplementary Table S4). The following characteristic features of samples were observed:

- **no microporosity** occurred in the chert samples SA3, SA8 and SA9!a;
- **all samples exhibit the majority of open pores in the mesopores regime** regardless of their position in the layer. Simultaneously nodular chert samples were characterized by pore space only in the range of mesopores (Fig. 6).
- sample **SA9 had the most micropores** (0.1%), sample **SA9!a had the most mesopores** (2.7%), and sample **SC2 had the most macropores** (0.1%);
- **differences in mean ratios of micro-**,** meso- and macropores can be found among outcrops**: 18% micropores, 78% mesopores and 4% macropores in Sowiniec Horst; 4.4% micropores, 93.9% mesopores and 1.7% macropores in Ujazd; 7.5% micropores, 89.7% mesopores and 2.8% macropores in Tyniec;
- **outcrops differed in terms of the open porosity PSD logarithmic means**: 12.1 nm, 10.2 nm and 10 nm for the Ujazd, Sowiniec Horst and Tyniec outcrops, respectively.

#### One-dimensional techniques: summary

In summary, based on one-dimensional data, significant differences were found between nodular and bedded cherts, inner and outer bedded chert layer parts and different outcrops. Many NMR parameters are directly correlated to the chemical composition, based on which it is impossible to directly differentiate outcrops as chemical information strongly determines visible lithological differences between samples. Therefore, such descriptive data interpretation is insufficient to roughly divide samples to their natural source location. However, some of the observations suggest subtle differences among outcrops related to the changes in open and closed porosity, bounding strengths of pore space, mean pore sizes and abundance of macro and micro-porosity. Despite that, a lot of obtained data is unrelated to outcrop differentiation and should be filtered out. Thus, 1D data sets have been considered in PCA.

### Outcrops differentiation based on two-dimensional techniques

#### Identification of hydrogen populations on *T*_*1*_-*T*_*2*_ correlation maps

This method delivered diversified *T*_*1*_-*T*_*2*_ correlation maps, where nine different peaks can be distinguished (Supplementary Table S5). The specific nature of the experiment allowed the association of each peak to a different hydrogen population. In the dry state, diversified signals can be seen. These may stem from chemically bound hydrogen, strongly adsorbed, or trapped water (which is unable to escape in the heating process, which can be observed for very tortuous pores) and inclusions, as hypothesized previously^12^.

##### Chemically bound hydrogen-hydroxyls

Chemical bonding in the form of hydroxyl groups (OH) is usually indicated by *T*_*2*_ = 0.06–0.2 ms and *T*_*1*_/*T*_*2*_ = 400–500,^52,53^ as in the case of characteristically elongated peaks 1 and 2. In silicates, OH bonds can be formed in silanols (O_silanol_–H_silanol_), between free water molecules (O_water_–H_water_) and between silanol^16^O and free water^1^H (O_silanol_– H_water_). The hydrogen bonding lengths in these pairs are equal to 0.92, 1.8 and 1.72 Å, respectively^54^. Higher values of *T*_*2*_ for peak 2 than for peak 1 with a preserved high value of *T*_*1*_/*T*_*2*_ ratio suggests weaker silanol hydrogen bonding strength. It was shown that a higher chemical shift was proportional to hydrogen bonding length^55^. Therefore, one possible scenario is that peak 1 comes from silanols, i.e., hydrogen chemically bound in the sample’s matrix, while peak 2 comes from O_silanol_– H_water_. The other scenario is that peak 1 comes from silanols from the deep matrix (structural OH), while peak 2 is from silanols on the open pores surface^56^. Because of similar *T*_*1*_/*T*_*2*_, those peaks characterize the covalent bonds rather than H-bonds with molecular water. These hypotheses can be transferred to the surface containing Ca(OH)_2_, where OH also might be formed between molecular water and calcium oxide. After water saturation, some samples were also significantly saturated in population S1/SD1 (Figs. 8 and 9), which indicates that new silanols can be formed in the saturation process.

**Fig. 7:**
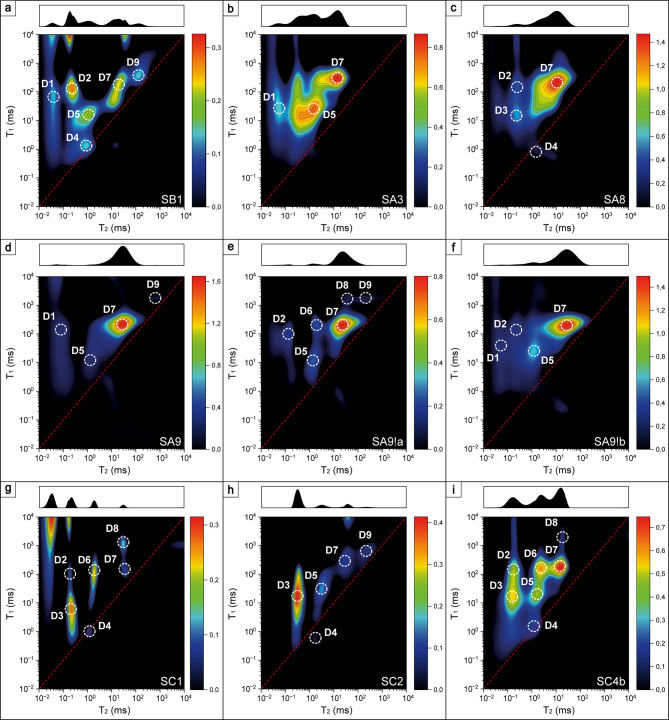
*T*_*1*_-T_*2*_ correlation maps of oven-dried samples.

**Fig. 8:**
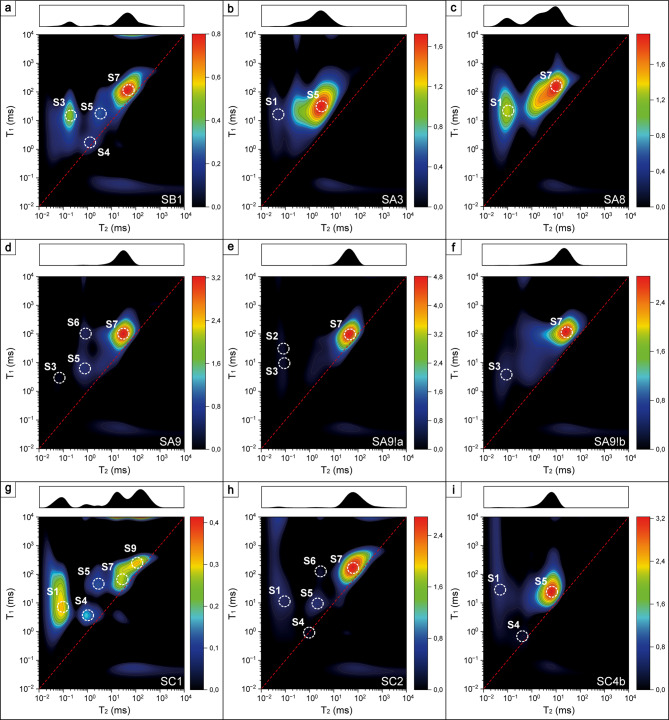
*T*_*1*_-T_*2*_ correlation maps of water-saturated samples.

##### Adsorbed/surface water

The reported signature signal of water adsorbed on the silica surface for silica glass was *T*_*2*_ ~ 1 ms and *T*_*1*_*/T*_*2*_ ~ 64, and for synthetic samples MCM-41 and SBA-15 was reported to be equal to *T*_*2*_ ~ 2 ms and *T*_*1*_*/T*_*2*_ ~ 7 and 45 for MCM-41 and SBA-15, respectively^53,56^. In this study, this population is represented by peak 6, especially since it was not visible in the saturated state, probably because its signal averaged with pore bulk water resolved as a single peak. Peak 3 also suits the adsorbed water criterion in terms of *T*_*2*_, having *T*_*1*_*/T*_*2*_ values in the range of 36–103. Slightly higher values suggest that peak 3 can also represent very small pores, where water is entrapped between pore walls and surface water relaxation dominates, especially since it is also visible on differential maps reflecting movable water.

##### Pore water

**Fig. 9:**
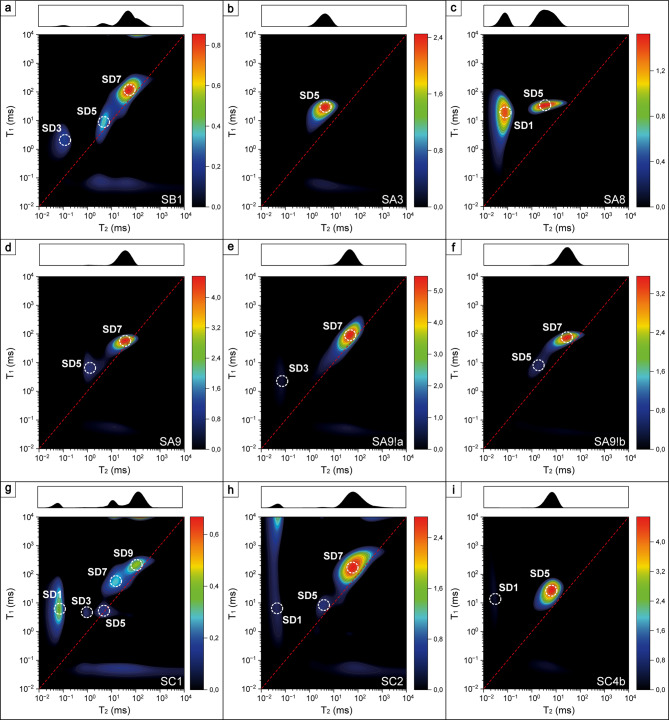
*T*_*1*_-T_*2*_ correlation maps of differential data from saturated and dry samples.

Based on the *T*_*1*_*/T*_*2*_ = 1.8–26 it can be suspected that despite *T*_*2*_ ~ 1 ms peak 5 comes from water entrapped in small pores rather than adsorbed water, and this signal is also visible as movable water (Fig. 9). Increased *T*_*1*_*/T*_*2*_ for these two peaks can result from the fact that in small pores higher relative number of molecules feels the attraction from the surface, and the concept is called layering^47^. Other peaks come from populations in pores in general (meaning that pore bulk water dominated), while the larger the *T*_*2*_ the larger the pore and the larger the *T*_*1*_/*T*_*2*_ the stronger immobilization of water in a pore. Those populations identified as pore water in the dry state came from inclusions.

After water saturation, some peaks disappeared from the maps, which can result from their low abundance in the whole porosity population and thus low signal intensity compared to the saturated regions (Fig.  8). Another hypothesis is that those disappeared hydrogen populations averaged with more abundant populations (for example, surface water with bulk water in a pore) and were not resolved as a separate peak. The most frequent samples were saturated in the regions represented by S3, S5, S7 and S9, which suggests that open porosity was associated with bigger pores.

#### Qualitative assessment of outcrops’ maps differences

In this two-dimensional experiment, some differences among outcrops can be noticed. In general, the obtained *T*_*2*_ values were in the range of ~ 0,02-1000 ms, with peaks ranging from 0.0398 to 589 ms (*T*_*1*_/*T*_*2*_ = 0,2-1739), 0.0398 to 105 ms (*T*_*1*_/*T*_*2*_ = 1-2138) and 0.0282 to 97.7 ms (*T*_*1*_/*T*_*2*_ = 1.15–535) for dry (Fig. 7), saturated (Fig. 8) and differential (Fig. 9) states, respectively (Supplementary Table S5). Dividing those ranges into outcrops, in:

- Sowiniec Horst: *T*_*2*_ = 0.0398-129 ms (*T*_*1*_/*T*_*2*_ = 1.32–1739), 0.182–42.7 ms (*T*_*1*_/*T*_*2*_ = 1.51-83) and 0.112-49 ms (*T*_*1*_/*T*_*2*_ = 1.86-20);
- Ujazd: *T*_*2*_ = 0.0525-589 ms (*T*_*1*_/*T*_*2*_ = 0.2–1739), 0.0457-39.8 ms (*T*_*1*_/*T*_*2*_ = 2.29–381) and 0.0646-45.7 ms (*T*_*1*_/*T*_*2*_ = 1.62–190);
- Tyniec: *T*_*2*_ = 0.158-209 ms (*T*_*1*_/*T*_*2*_ = 0.31–659), 0.0398-105 ms (*T*_*1*_/*T*_*2*_ = 1-2138) and 0.0282-97.7 ms (*T*_*1*_/*T*_*2*_ = 1.15–535),

for the dry, saturated and differential states, respectively. The differences are mostly visible on the maps obtained for dry samples, especially on the *T*_*2*_ profiles of the maps. SB1 has a wide profile with no specifically distinguished peaks, Ujazd samples are dominated by the peak D7, and for nodular cherts shorter *T*_*2*_ peaks are marked, Tyniec samples had multiple well-separated peaks. Outcrops can also be ordered from having the widest *T*_*2*_ profile and largest *T*_*1*_/*T*_*2*_ of open (differential state) and total (saturated state) porosity as follows: Tyniec, Ujazd and Sowiniec Horst.

#### Two-dimensional techniques: summary

*T*_*1*_*/T*_*2*_ correlation maps emphasized differences among outcrops based on the open and total porosity, but also subtle differences in chemically bound hydrogen populations and inclusions. Such an experiment better resolves the complex system of hydrogen species, which can vary due to many factors depending on the different courses of silicification processes in individual calciturbidite outcrops. Due to the complexity of the maps and the specificity of the 2D Laplace Transform it cannot be clearly stated if the differences are sufficient to resolve between outcrops or they rather resulted from ambiguity of the transformation. Therefore, 2D data sets were also subjected to PCA analysis to reduce information noise and leave only statistically significant data from the perspective of outcrop differentiation.

### Principal component analysis (PCA)

#### Assessment of the outcrop separation ability of different datasets

**Fig. 10:**
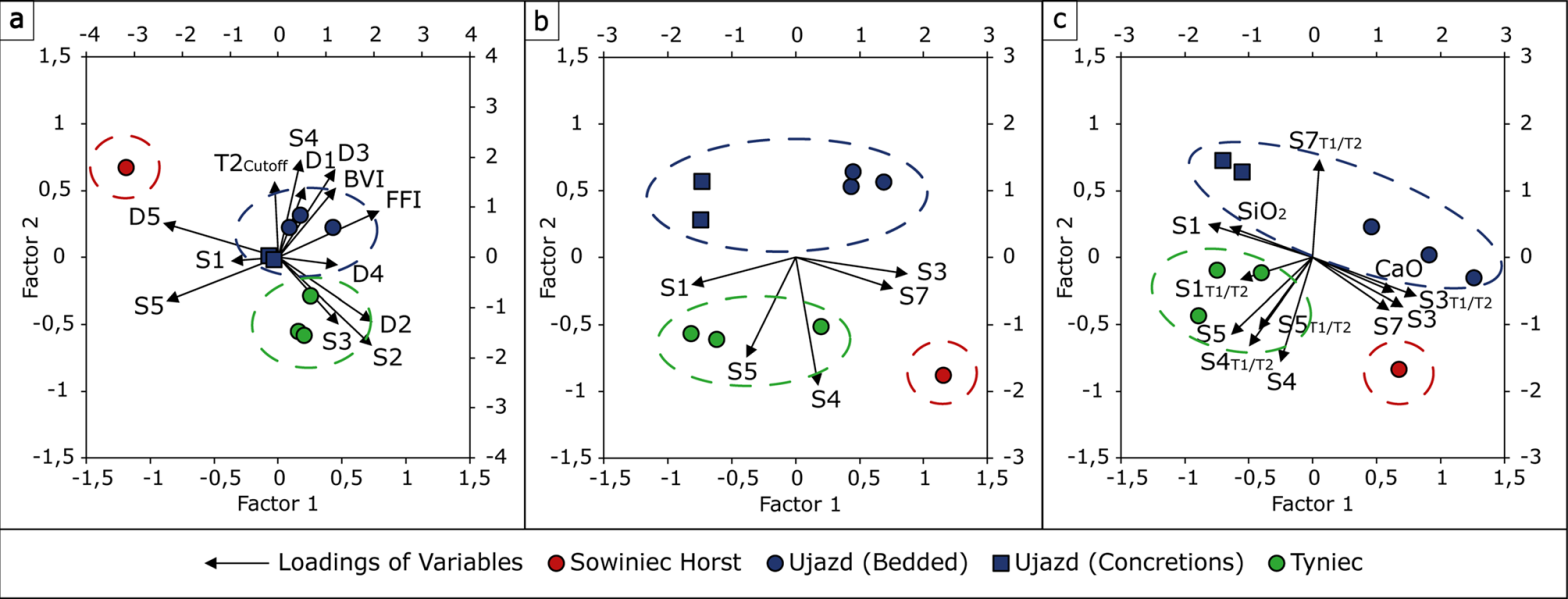
PCA analysis for different sets of primary variables: 1D-*T*_*2*_ NMR data including *T*_*2*_ times of peaks from distributions of dry (D1, D2, D3, D4, D5) and saturated (S1, S2, S3, S4, S5) samples, and *T*_*2*_ standard protocol data: *T*_*2cutoff*_, BVI, and FFI (**a**); only *T*_*2*_ times of peaks from 1D-*T*_*2*_ distributions of saturated (S1, S2, S3, S4, S5) samples (**b**); 2D *T*_*1*_*-T*_*2*_ maps data including *T*_*2*_ times of peaks from maps obtained for saturated samples (S1, S2, S3, S4, S5) and *T*_*1*_/*T*_*2*_ ratios for these peaks, as well as SiO_2_ and CaO content (**c**).

The differences that were found in the analysis of the chemical composition and NMR data did not provide sufficient evidence to conclude that there is a certain feature of the samples that determines the outcrop. Hence, the statistical analysis was made by using PCA. As shown in the workflow, first the basic parameters obtained from individual experiments were tested, and then the most different features were combined to check how they would affect the result of the PCA analysis. Basic parameters describing a rock core sample such as chemical composition, and if NMR is used, petrophysical quantities such as BVI or FFI fail in separating outcrops (Supplementary Figure S5). In the next step, more detailed NMR parameters were tested in combined sets, which reflect comprehensive information about the samples related to their chemical structure, porosity system and pore surface features. The results are shown in Fig. 10 and the primary observations are:

- ***T***_***2cutoff***_, ***T***_***2***_**times and porosities from dry and saturated samples were capable of resolving three outcrops** (1D *T*_*2*_ times alone did not deliver the expected results), but groups are close to each other, and this may lead to incorrect grouping by an independent tester (for example one might classify Tyniec and Ujazd as a single outcrop);
- ***T***_***2***_**times from the*T***_***1***_**-*T***_***2***_**correlation experiment of saturated samples showed an excellent outcrop separation ability through “hydroxyls features” and “pores features”**,** respectively** (Factor 1 connects S1 associated with structural OH bonding and S3 associated with surface water adsorbed physically or chemically through H-bonds, while Factor 2 S4 and S5 peaks associated with pore water);
- **combined set of the abovementioned*T***_***2***_**times**, ***T***_***1***_**/*T***_***2***_**ratios and SiO**_**2**_**and CaO content preserved the earlier outcrop separation** and introduced stronger Tyniec samples clustering and Ujazd samples collinearity;
- **using a dataset with*Fe***_***2***_***O***_***3***_***/TiO***_***2***_**and*Al***_***2***_***O***_***3***_***/(Al***_***2***_***O***_***3***_ ***+ Fe***_***2***_***O***_***3***_***)*factors did not introduce any visible change of separation**. As indicated in Sect. 3, almost all samples were related to the same environment of deposition - Ocean Ridge with an influence of hydrothermal activity in the silicification process. Therefore, the introduction of chemical tectonic-setting parameters into the PCA analysis did not yield new results in comparison to the approach with SiO_2_ and CaO content (Supplementary Figure S5c).

#### Relationships between NMR parameters and sample micro-characteristics

PCA analysis revealed and clarified relationships between NMR parameters and sample characteristics related to chemical structure and porosity features:

- S3 and S7 were strongly correlated, so **there was a correlation between*T***_***2***_**of surface and pore water populations**, which means that they may come from the same pore type (e.g. in terms of size), where surface relaxation dominates the pore relaxation time;
- SiO_2_ was positively correlated with *T*_*2*_ of S1 and CaO was positively correlated with *T*_*2*_ of S3, which together with individual samples occurrence indicates that ***T***_***2***_**of structure OH groups was sensitive to the degree of silicification and crystallinity index** equal to around 2.3, 5 for bedded cherts from Ujazd and Tyniec samples and 6–7 for nodular cherts^2^, respectively. More crystalline samples were characterized by lower *T*_*2*_ of OH in silanols (i.e. OH covalent bonding), which shifted towards higher values in more amorphous systems.
- Factor 2 in Fig. 10b connected to the ***T***_***2***_**of water in pores S4 and S5 discriminated the samples with different calciturbidite host rock primary structure characteristics**: These open pore populations occurred mainly for Sowiniec Horst and Tyniec outcrops. for Ujazd cherts this populations were lower or did not occur at all;
- the correlation of S3 and S7 with CaO and the cluster of bedded cherts from different outcrops and no correlation between *T*_*2*_ and *T*_*1*_/*T*_*2*_ of peak S7 indicated that **the size of open porosity was mainly dependent on the CaO content**,** but the pore surface characteristics of those pores depended on the primary depositional structures of carbonate host-rock** and thus also separated the Ujazd outcrop from the rest.

#### Relationships between NMR parameters and outcrops

PCA analysis highlighted differences in NMR parameters that contributed most to the differentiation of outcrops. It should be emphasized that it was possible to distinguish outcrops not thanks to certain parameters, but to the accumulated information about the sample contained in the entire dataset. Some features were found that together with the observations provided in Sect. 3.1 and 3.2 made it possible to divide outcrops. These features are:

- Tyniec samples evinced the capability to form new silanols after the water saturation (S1 appeared only after water saturation);
- Tyniec samples had residual surface water in the dry state (peak D3);
- Ujazd samples were characterized by the strongest water absorption in pores S5 and S7 (based on *T*_*1*_/*T*_*2*_), with the highest adsorption energy for nodular cherts;
- Ujazd samples lacked open porosity represented by population S4 and had the smallest pores in population S5;
- Sowiniec Horst sample separates from others based on the biggest open pores (based on Fig. 10b and c sample lies between vectors representing S3, S4 and S7);
- Sowiniec Horst sample had the strongest OH bonds;
- Sowiniec Horst sample had the largest amount of signal interpreted as inclusions (peak D9);
- Sowiniec Horst sample had the largest surface roughness in the population represented by peak 5.

## Conclusions

Simple chemical composition data and different macroscopic features are insufficient to distinguish between cherts samples. In the study, we showed that LF-NMR results reflect complex information about porous space including closed (inclusions) and open porosity, rock-matrix chemical structure and geological features such as structures of carbonate host rock or position of sample in bedded chert layer. In most cases single NMR parameter represents a mix of those features. However, some subtle differences in NMR parameters were emphasized in the statistical analysis made by PCA. Our complex analysis helped to characterize samples and indicated the features common to their position in the bedded chert layer, primary depositional structures of carbonate host-rock, silicification type and finally – source outcrop. This objective approach for the classification of siliceous rocks can be further tested on a larger set and help to enhance the sometimes-subjective archaeological studies of siliceous artefacts not only from the KCU region but also from other regions of natural chert outcrops occurrence.

## Electronic supplementary material

Below is the link to the electronic supplementary material.

Supplementary Material 1

## Data Availability

Data used to reach the conclusion of this study is presented in the paper and the Supplementary Materials. Source data is provided via https://doi.org/10.17632/5bcym49f67.2 at Mendeley Data repository with CC BY 4.0 license.
