# Supplementary material for: Chert outcrops differentiation by means of low-field NMR relaxometry
Source: Sci Rep. 2024 Oct 25;14:25280. doi: 10.1038/s41598-024-75945-6 (PMC11512048; doi:10.1038/s41598-024-75945-6)
Supplement: Supplementary file 1 — Supplementary Material 1 [file 41598_2024_75945_MOESM1_ESM.pdf]

Chert Outcrops differentiation by means of Low-Field NMR Relaxometry

Michał Fajt[1], *Weronika Mazur-Rosmus*[1], *Anna Stefańska*[1], *Alicja Kochman*[1], *Artur T. Krzyżak*[1]

[1]Faculty of Geology, Geophysics and Environmental Protection, AGH University of Krakow, al. Adama Mickiewicza 30, 30-059 Krakow, Poland.

Supplementary Information

Table of Contents

Tables

Supplementary Table S1. Major element contents (%) obtained by FUS-ICP in the bedded and nodular cherts. S3

Supplementary Table S2. T1 relaxation times of peaks from T1 distributions of dry, saturated and differential data of investigated KCU nodular and bedded chert samples. S4

Supplementary Table S3. Standard LF-NMR protocol porosity determination results: cut-off time ($T_{2cutoff}$) bulk-volume irreducible (*BVI*), free-fluid index (*FFI*), saturated water irreducible (*SWI*) and Total Porosity with mass-volume measurements results and calculated surface relaxivity ρ_2 for all investigated chert samples. S5

Supplementary Table S4. T_2 relaxation times of peaks from T_2 distributions and determined NMR parameters of dry, saturated and differential data investigated KCU nodular and bedded chert samples. d_{mean} – mean pore diameter, ϕ_{meso} – mesoporosity, ϕ_{macro} – macroporosity, ϕ_i – cumulative porosity (a sum of meso- and macro-porosity)........ S6

Supplementary Table S5. The coordinates and T_1/T_2 coefficients for peaks distinguished and marked in Figures 4-6. .... S7

Figures

Supplementary Figure S1. Results of regression analysis. Logistic function fitted to literature values of diffusion coefficient... S8

Supplementary Figure S2. T_1 distributions of dry (first column), saturated (second column) and differential (third column) data of investigated chert samples with the division to three outcrops: Sowiniec Horst (sample SB1, panels a-c), Ujazd (samples SA3, SA8, SA9, SA9!a, SA9!b, panels d-f) and Wielkanoc Quarry (samples SC1, SC2, SC4b, panels g-i)... S9

Supplementary Figure S3. Discrimination diagrams for the major oxides showing the tectonic regions of chert samples formation.[34] S10

Supplementary Figure S4. Comparison between PSDs estimated assuming diffusion influence and without this correction, calculated by omitting diffusion component in equation 7. .... S11

Supplementary Figure S5. PCA analysis for different sets of primary variables: a – All chemical components of the samples; b – All chemical components of the samples with standard T_2 analysis protocol data ($T_{2cutoff}$, bulk volume irreducible, BVI, free fluid volume, FFI); c – 2D T_1-T_2 maps data including T_2 times of peaks from maps obtained for saturated samples (S1, S2, S3, S4, S5) and T_1/T_2 ratios for these peaks, as well as Fe_2O_3/TiO_2 and $Al_2O_3/(Al_2O_3+Fe_2O_3)$ factors. S12

Supplementary Figure S6. Cross-validation of mass-volume porosity and corresponding NMR open porosity derived from differential distributions. S12

Supplementary Table S1. Major element contents (%) obtained by FUS-ICP in the bedded and nodular cherts.

Sample	Outcrop	Host rock	SiO_2	Fe_2O_3	CaO	Al_2O_3	MnO	MgO	Na_2O	K_2O	TiO_2	P_2O_5	LOI
**SB1**	Sowiniec Horst	calciturbidites	92,76	0,61	3,58	0,19	0,009	0,04	0,04	0,03	0,005	0,02	2,81
**SA3**	Ujazd	beneath a layer of bedded chert in calciturbidites	99,02	1,03	0,10	0,12	0,010	0,01	0,05	0,03	0,003	< 0,01	0,38
**SA8**		above a layer of bedded chert in calciturbidites	97,94	0,96	0,05	0,16	0,010	0,01	0,06	0,04	0,005	< 0,01	0,75
**SA9**		calciturbidites	69,65	0,21	16,94	0,12	0,009	0,14	0,03	0,03	0,004	0,02	13,43
**SA9!a**		calciturbidites	58,74	0,14	23,05	0,12	0,011	0,21	0,03	0,03	0,004	0,04	18,08
**SA9!b**		calciturbidites	91,17	0,74	4,05	0,16	0,010	0,04	0,04	0,03	0,005	0,03	3,85
**SC1**	Tyniec	calciturbidites	87,11	0,52	7,08	0,09	0,009	0,06	0,03	0,01	0,003	0,02	5,37
**SC2**		calciturbidites	83,47	0,48	9,32	0,14	0,011	0,08	0,03	0,02	0,003	0,02	7,11
**SC4b**		calciturbidites	86,44	0,63	7,14	0,13	0,010	0,07	0,04	0,03	0,004	0,03	5,81

Supplementary Table S2. T1 relaxation times of peaks from T1 distributions of dry, saturated and differential data of investigated KCU nodular and bedded chert samples.

**Sample**	**Peak number**								**T_{1lm} (ms)**
	**T_1 Relaxation Time (ms)**								
**Dry**	**d1**	**d2**	**d3**	**d4**	**d5**	**d6**	**d7**	**d8**	
**SB1**	0,061	0,60		16,8		581			16,4
**SA3**	0,075	0,46	5,54	25,5	204,9			4055	38,5
**SA8**	0,017	1,70		22,2	89,1	311			28,6
**SA9**		0,60	4,50	22,2	166,4	581			90,7
**SA9!a**			2,97	41,5	144,8	622			87
**SA9!b**		0,56	2,97	22,2	144,8	581			72,8
**SC1**	0,065		4,50	13,7		290			3,27
**SC2**	0,037	1,96		16,8	135,1		1012		2,29
**SC4b**		0,98		16,8	117,6	357			45,6
**Saturated**	**s1**	**s2**	**s3**	**s4**	**s5**	**s6**	**s7**	**s8**	
**SB1**	0,075		5,54	23,8	109,7	542			41,3
**SA3**		3,41		25,5	166,4	542			39,9
**SA8**	0,028	2,10	12,75		54,8	290			47,9
**SA9**		2,25		20,7	109,7	505			67,7
**SA9!a**		2,58		25,5	117,6	410			79,6
**SA9!b**		1,20	11,90		62,9	191			82,8
**SC1**		2,10		41,5		581			97,8
**SC2**		3,18		33,7	109,7	333			130
**SC4b**		4,20	11,10	31,4	191,2				31,5
**Differential**	**sd1**	**sd2**	**sd3**	**sd4**	**sd5**	**sd6**	**sd7**	**sd8**	
**SB1**			5,94		109,7				52,8
**SA3**		3,18		22,2	83,1				27,4
**SA8**	0,122		5,54	36,1	54,8				20
**SA9**		1,38	9,66		58,7	471			39,6
**SA9!a**		1,83		19,3	77,5	220			68,7
**SA9!b**			9,01		67,5	191			72,6
**SC1**		1,29		44,5		471			95,3
**SC2**		3,18		29,3	67,5	270			140
**SC4b**			6,37	29,3	109,7				24,6

Supplementary Table S3. Standard LF-NMR protocol porosity determination results: cut-off time ($T_{2cutoff}$) bulk-volume irreducible (*BVI*), free-fluid index (*FFI*), saturated water irreducible (*SWI*) and Total Porosity with mass-volume measurements results and calculated surface relaxivity ρ_2 for all investigated chert samples.

Sample	$T_{2cutoff}$ (ms)	*BVI* (%)	*FFI* (%)	Total Porosity (%)	*SWI* (-)	m_{sat} (g)	m_{dry} (g)	V_{sample} (cm^3)	$\phi_{mass\text{-}vol}$ (%)	ρ_2 (µm/s)
**SB1**	13,7	0,3	0,3	0,6	0,50	56,60	56,51	21,99	0,4	0.10
**SA3**	3,7	1,2	0,5	1,7	0,71	60,34	60,22	23,46	0,5	0.44
**SA8**	19,3	1,6	0,1	1,7	0,94	56,65	56,62	21,92	0,2	0.27
**SA9**	20,7	1,0	0,8	1,8	0,56	54,11	53,92	21,60	0,9	0.11
**SA9!a**	11,9	0,7	2,8	3,5	0,20	32,28	31,91	12,95	2,8	0.08
**SA9!b**	16,8	1,3	0,8	2,1	0,62	53,87	53,73	21,25	0,7	0.16
**SC1**	7,3	0,1	0,4	0,5	0,20	31,17	31,11	12,10	0,5	0.05
**SC2**	7,8	0,2	1,3	1,5	0,13	59,21	58,86	23,20	1,5	0.04
**SC4b**	3,2	1,2	1,7	2,9	0,41	38,79	38,56	15,02	1,6	0.26

Supplementary Table S4. T_2 relaxation times of peaks from T_2 distributions and determined NMR parameters of dry, saturated and differential data investigated KCU nodular and bedded chert samples. d_{mean} – mean pore diameter, ϕ_{meso} – mesoporosity, ϕ_{macro} – macroporosity, ϕ_i – cumulative porosity (a sum of meso- and macro-porosity).

**Samples in a given saturation state**	**T_2 relaxation times of peaks (ms)**					**T_{2lm} (ms)**	**T_{1lm}/T_{2lm} (-)**	**d_{mean} (nm)**	**Φ_{micro} (%)**	**φ_{meso} (%)**	**φ_{macro} (%)**	**ϕ_i (%)**
**Dry**	**D1**	**D2**	**D3**	**D4**	**D5**							
**SB1**	0,455		7,84	47,7	178,3	2,85	0,27					0,27
**SA3**	0,425	5,54	31,4			1,05	1,22					1,22
**SA8**	0,488	6,37				2,30	1,59					1,59
**SA9**	0,455	4,82	27,4	126,0		8,03	0,95					0,95
**SA9!a**	0,691	7,32	31,4	126,0		12,1	0,67					0,67
**SA9!b**	0,370	4,20	23,8	102,3		3,53	1,31					1,31
**SC1**	0,370	6,83		95,5		1,13	0,14					0,14
**SC2**	0,345	7,32		83,1		1,49	0,17					0,17
**SC4b**	0,370	4,82	22,2	135,1		1,12	1,19					1,19
**Saturated**	**S1**	**S2**	**S3**	**S4**	**S5**							
**SB1**	0,793		13,67	54,8	333,1	9,84	0,57					0,57
**SA3**	0,793	4,50				1,43	1,73					1,73
**SA8**	0,523	5,17	20,7			2,85	1,74					1,74
**SA9**	0,601	4,82	29,3	117,6		12,8	1,77					1,77
**SA9!a**	0,793	6,37	36,1			25,9	3,45					3,45
**SA9!b**	0,370	3,65	25,5	109,7		5,21	2,07					2,07
**SC1**	0,691	7,32	33,7		178,3	18,7	0,49					0,49
**SC2**	0,601	7,32	41,5		166,4	43,5	1,48					1,48
**SC4b**	0,601	6,83	38,7			3,14	2,83					2,83
**Differential**	**SD1**	**SD2**	**SD3**	**SD4**	**SD5**							
**SB1**	2,58		19,3	72,3	383	25,0	0,31	12.1	0.06	0.24	0.01	0,31
**SA3**	2,41	8,41				3,60	0,50	6.9		0.50		0,50
**SA8**	3,41		19,3			5,64	0,21	8.5		0.21		0,21
**SA9**	2,25		33,7			19,3	0,85	10.6	0.10	0.75		0,85
**SA9!a**	3,41		19,3	58,7	311	32,8	2,75	13.2		2.72	0.03	2,75
**SA9!b**	3,41		25,5	83,1		10,5	0,75	11.5	0.08	0.62	0.05	0,75
**SC1**		5,94	33,7		191	41,6	0,36	9.4	0.05	0.31		0,36
**SC2**	2,41		38,7		178	59,8	1,34	14.7	0.05	1.18	0.11	1,34
**SC4b**	1,96	7,84				5,23	1,73	6.0	0.07	1.66		1,73

Supplementary Table S5. The coordinates and T_1/T_2 coefficients for peaks distinguished and marked in Figures 4-6.

Time Range (ms)	T_2~0,05 T_1~20		T_2~0,1 T_1~100		T_2~0,1 T_1~10		T_2~1 T_1~1		T_2~1 T_1~10		T_2~1 T_1~100		T_2~20 T_1~200		T_2~20 T_1~300-1600		T_2~130-200 T_1~400-2000	
**Sample**	T_2 (ms)	T_1/T_2	T_2 (ms)	T_1/T_2	T_2 (ms)	T_1/T_2	T_2 (ms)	T_1/T_2	T_2 (ms)	T_1/T_2	T_2 (ms)	T_1/T_2	T_2 (ms)	T_1/T_2	T_2 (ms)	T_1/T_2	T_2 (ms)	T_1/T_2
**Dry**	**D1**		**D2**		**D3**		**D4**		**D5**		**D6**		**D7**		**D8**		**D9**	
**SB1**	0,040	1739	0,224	576			0,832	1,32	1,1	14			18,6	9,78			129	2,81
**SA3**	0,052	467							1,45	1,81			14,1	21				
**SA8**			0,24	538	0,224	51	1,45	0,57					10,7	20				
**SA9**	0,052	617					7,08	0,20	1,66	18			28,2	8,51			589	1,69
**SA9!a**			0,129	868					1,35	9,11	1,91	72	22,9	10	34,7	24	170	12
**SA9!b**	0,056	381	0,085	1739					1,17	26			28,2	8,51				
**SC1**			0,182	659	0,209	36	1,17	0,82			1,78	78	32,4	6,02	28,2	59		
**SC2**					0,316	68	1,66	0,31	3,09	12			28,2	15			209	3,98
**SC4b**			0,17	467	0,158	103	1,26	1,62	1,66	17	2,34	59	15,1	17	2,34	103		
**Saturated**	**S1**		**S2**		**S3**		**S4**		**S5**		**S6**		**S7**		**S8**		**S9**	
**SB1**					0,182	83	1,1	1,51	3,09	6,02			42,7	2,62				
**SA3**	0,045	381							2,88	10								
**SA8**	0,091	166											9,33	13				
**SA9**					0,064	42			0,631	9,78	0,776	135	30,2	3,24				
**SA9!a**			0,074	178	0,079	48							39,8	2,29				
**SA9!b**					0,074	48							24,5	4,57				
**SC1**	0,085	78					0,891	3,98	2,69	15			22,9	2,45			105	2,29
**SC2**	0,052	501					0,832	1	1,78	4,89	2,69	39	60,3	2,82				
**SC4b**	0,039	2138					0,479	1,41	6,17	3,97								
**Differential**	**SD1**		**SD2**		**SD3**		**SD4**		**SD5**		**SD6**		**SD7**		**SD8**		**SD9**	
**SB1**					0,112	20			4,68	1,86			49	2,29				
**SA3**									4,07	6,93								
**SA8**	0,074	190							3,55	6,90								
**SA9**									1,1	5,61			34,7	1,62				
**SA9!a**					0,064	42							45,7	2,00				
**SA9!b**									1,78	4,26			28,2	2,63				
**SC1**	0,064	89			0,832	5,25			4,37	1,15			14,1	3,72			97,7	2,14
**SC2**	0,042	204							3,31	2,46			60,3	2,82				
**SC4b**	0,028	535							6,61	4,27								

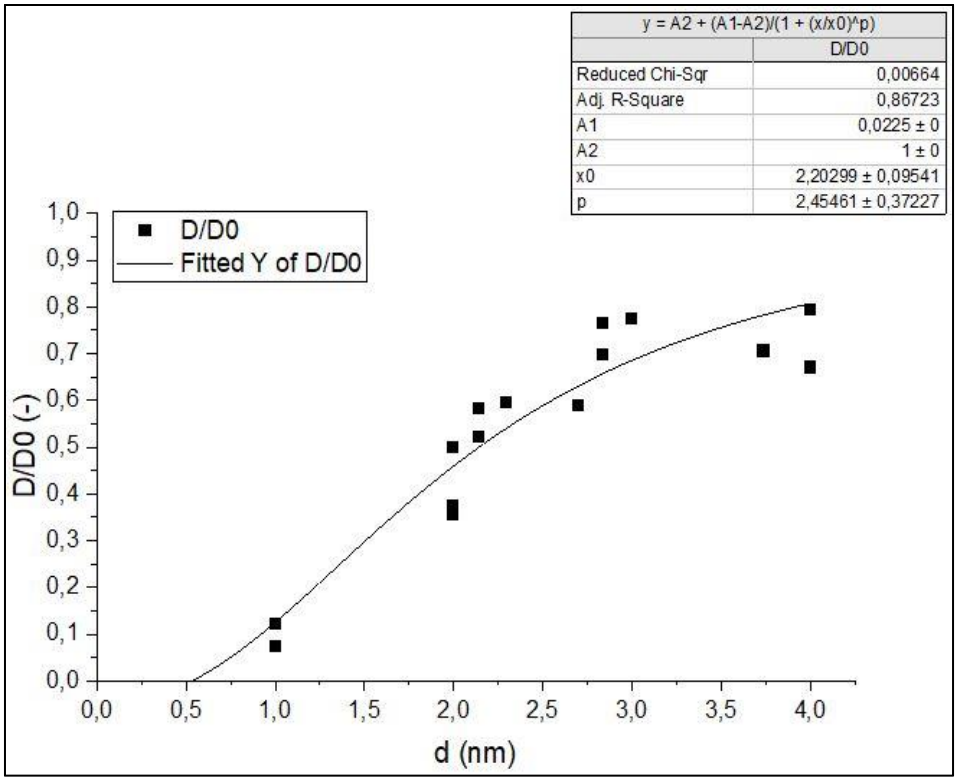

Supplementary Figure S1. Results of regression analysis. Logistic function fitted to literature values of diffusion coefficient

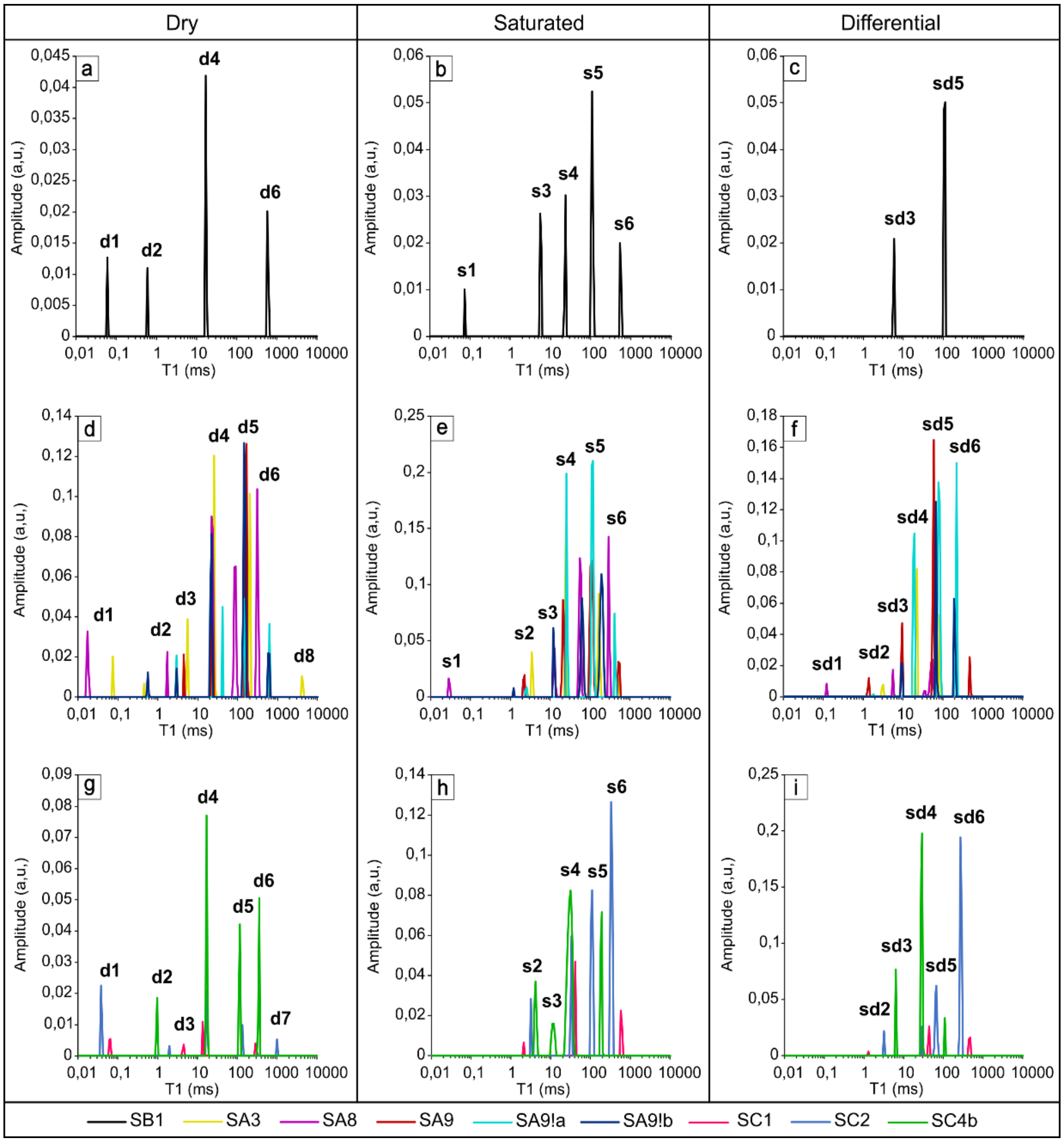

Supplementary Figure S2. T_1 distributions of dry (first column), saturated (second column) and differential (third column) data of investigated chert samples with the division to three outcrops: Sowiniec Horst (sample SB1, panels a-c), Ujazd (samples SA3, SA8, SA9, SA9!a, SA9!b, panels d-f) and Wielkanoc Quarry (samples SC1, SC2, SC4b, panels g-i).

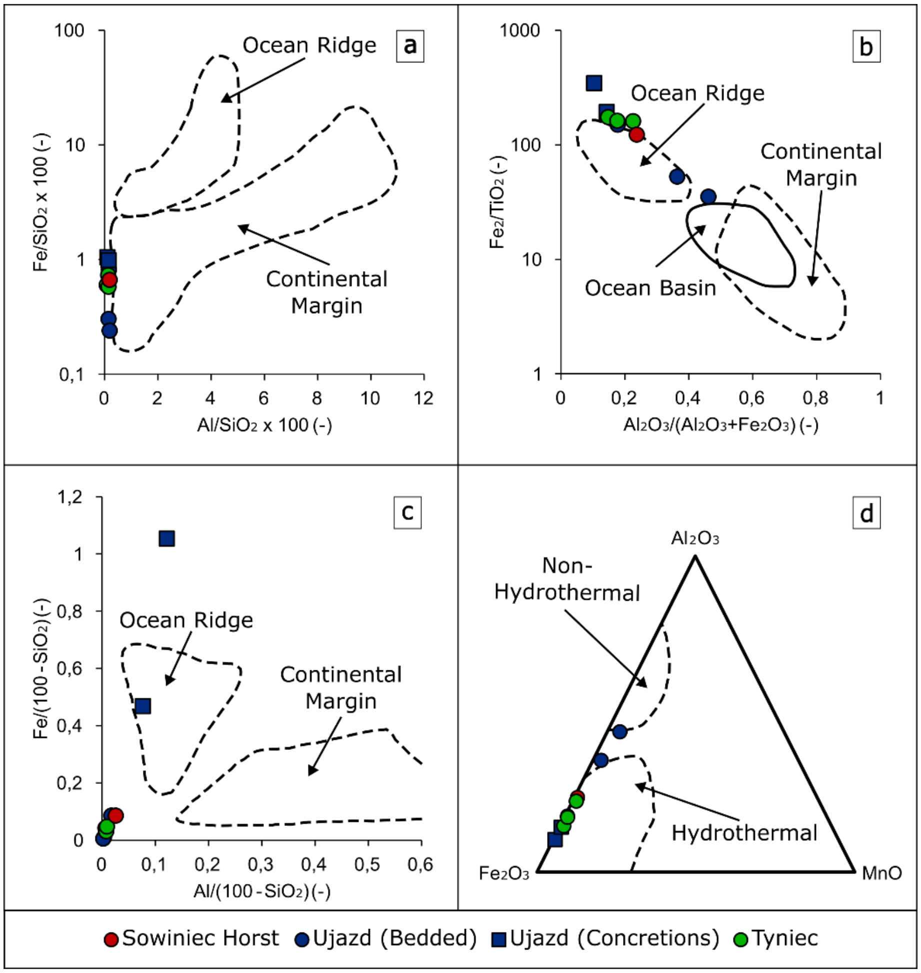

Supplementary Figure S3. Discrimination diagrams for the major oxides showing the tectonic regions of chert samples formation.[34]

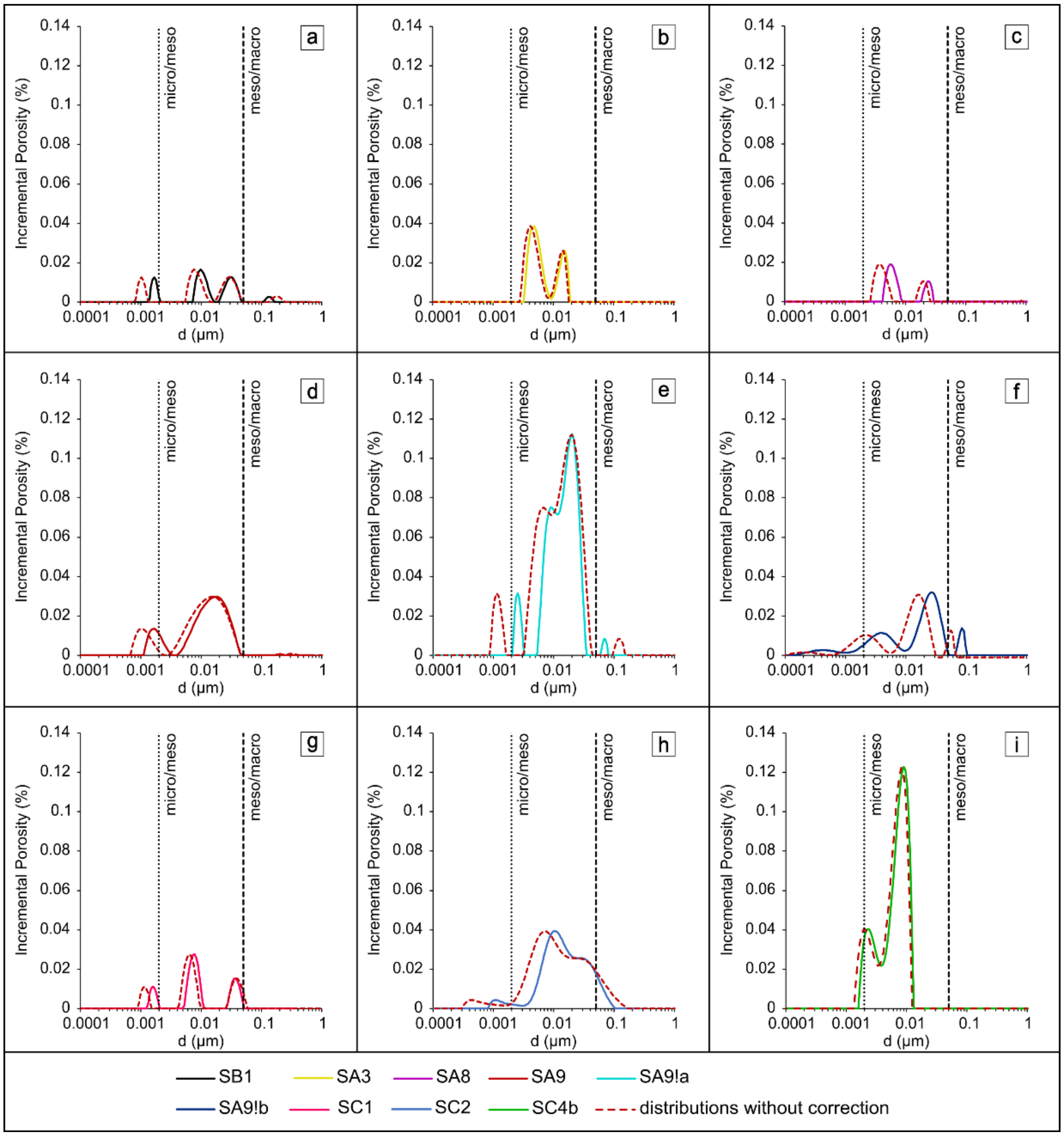

Supplementary Figure S4. Comparison between PSDs estimated assuming diffusion influence and without this correction, calculated by omitting diffusion component in equation 7.

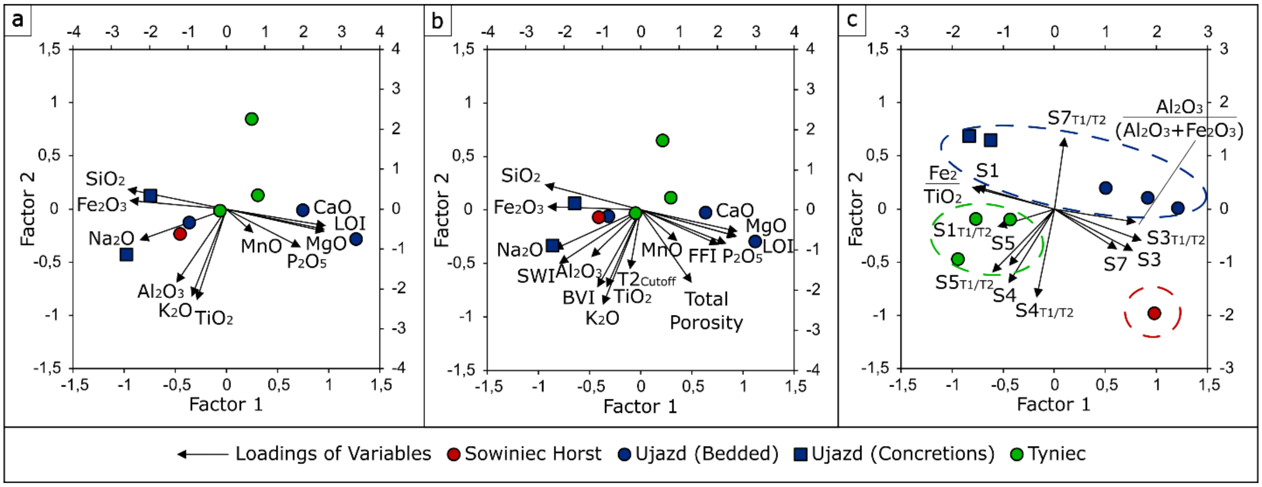

Supplementary Figure S5. PCA analysis for different sets of primary variables: a – All chemical components of the samples; b – All chemical components of the samples with standard T_2 analysis protocol data ($T_{2cutoff}$, bulk volume irreducible, BVI, free fluid volume, FFI); c – 2D T_1-T_2 maps data including T_2 times of peaks from maps obtained for saturated samples (S1, S2, S3, S4, S5) and T_1/T_2 ratios for these peaks, as well as Fe_2O_3/TiO_2 and $Al_2O_3/(Al_2O_3+Fe_2O_3)$ factors.

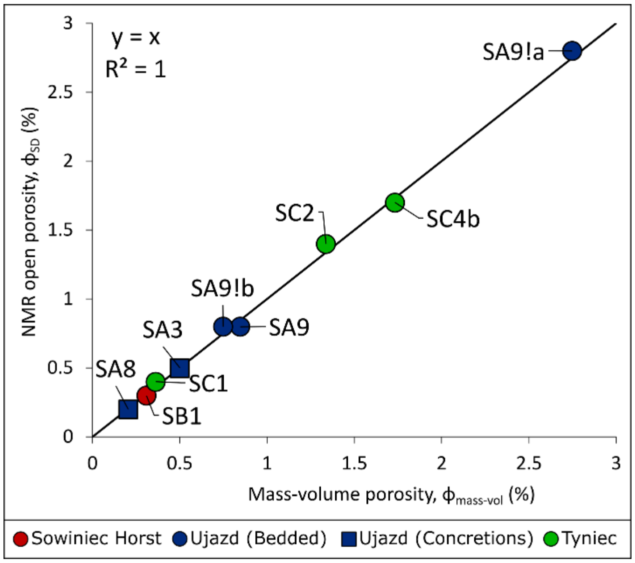

Supplementary Figure S6. Cross-validation of mass-volume porosity and corresponding NMR open porosity derived from differential distributions.
